# Air-quality-related health impacts from climate change and from adaptation of cooling demand for buildings in the eastern United States: An interdisciplinary modeling study

**DOI:** 10.1371/journal.pmed.1002599

**Published:** 2018-07-03

**Authors:** David W. Abel, Tracey Holloway, Monica Harkey, Paul Meier, Doug Ahl, Vijay S. Limaye, Jonathan A. Patz

**Affiliations:** 1 Center for Sustainability and the Global Environment (SAGE), Nelson Institute for Environmental Studies, University of Wisconsin–Madison, Madison, Wisconsin, United States of America; 2 Department of Atmospheric and Oceanic Sciences, University of Wisconsin–Madison, Madison, Wisconsin, United States of America; 3 Wisconsin Energy Institute (WEI), University of Wisconsin–Madison, Madison, Wisconsin, United States of America; 4 Great Lakes Bioenergy Research Center (GLBRC), University of Wisconsin–Madison, Madison, Wisconsin, United States of America; 5 Meier Engineering Research LLC, Stoughton, Wisconsin, United States of America; 6 Seventhwave, Madison, Wisconsin, United States of America; 7 Global Health Institute, University of Wisconsin–Madison, Madison, Wisconsin, United States of America; Africa Program, UNITED STATES

## Abstract

**Background:**

Climate change negatively impacts human health through heat stress and exposure to worsened air pollution, amongst other pathways. Indoor use of air conditioning can be an effective strategy to reduce heat exposure. However, increased air conditioning use increases emissions of air pollutants from power plants, in turn worsening air quality and human health impacts. We used an interdisciplinary linked model system to quantify the impacts of heat-driven adaptation through building cooling demand on air-quality-related health outcomes in a representative mid-century climate scenario.

**Methods and findings:**

We used a modeling system that included downscaling historical and future climate data with the Weather Research and Forecasting (WRF) model, simulating building electricity demand using the Regional Building Energy Simulation System (RBESS), simulating power sector production and emissions using MyPower, simulating ambient air quality using the Community Multiscale Air Quality (CMAQ) model, and calculating the incidence of adverse health outcomes using the Environmental Benefits Mapping and Analysis Program (BenMAP). We performed simulations for a representative present-day climate scenario and 2 representative mid-century climate scenarios, with and without exacerbated power sector emissions from adaptation in building energy use. We find that by mid-century, climate change alone can increase fine particulate matter (PM_2.5_) concentrations by 58.6% (2.50 μg/m^3^) and ozone (O_3_) by 14.9% (8.06 parts per billion by volume [ppbv]) for the month of July. A larger change is found when comparing the present day to the combined impact of climate change and increased building energy use, where PM_2.5_ increases 61.1% (2.60 μg/m^3^) and O_3_ increases 15.9% (8.64 ppbv). Therefore, 3.8% of the total increase in PM_2.5_ and 6.7% of the total increase in O_3_ is attributable to adaptive behavior (extra air conditioning use). Health impacts assessment finds that for a mid-century climate change scenario (with adaptation), annual PM_2.5_-related adult mortality increases by 13,547 deaths (14 concentration–response functions with mean incidence range of 1,320 to 26,481, approximately US$126 billion cost) and annual O_3_-related adult mortality increases by 3,514 deaths (3 functions with mean incidence range of 2,175 to 4,920, approximately US$32.5 billion cost), calculated as a 3-month summer estimate based on July modeling. Air conditioning adaptation accounts for 654 (range of 87 to 1,245) of the PM_2.5_-related deaths (approximately US$6 billion cost, a 4.8% increase above climate change impacts alone) and 315 (range of 198 to 438) of the O_3_-related deaths (approximately US$3 billion cost, an 8.7% increase above climate change impacts alone). Limitations of this study include modeling only a single month, based on 1 model-year of future climate simulations. As a result, we do not project the future, but rather describe the potential damages from interactions arising between climate, energy use, and air quality.

**Conclusions:**

This study examines the contribution of future air-pollution-related health damages that are caused by the power sector through heat-driven air conditioning adaptation in buildings. Results show that without intervention, approximately 5%–9% of exacerbated air-pollution-related mortality will be due to increases in power sector emissions from heat-driven building electricity demand. This analysis highlights the need for cleaner energy sources, energy efficiency, and energy conservation to meet our growing dependence on building cooling systems and simultaneously mitigate climate change.

## Abstract 2

David Abel and colleagues predict future mortality associated with increased emissions from power plants caused by demand for air conditioning in a warmer environment.

## Author summary

**Why was this study done?:**

- As temperature rises, the adaptive response of using air conditioning increases electricity demand and subsequent emissions of harmful pollutants from electric power plants.
- Independent of emissions from the electric power sector, climate change is known to worsen air quality through changes in atmospheric chemistry and natural biogenic emissions.
- We examine the effects of climate change on air quality and human health through the combined impacts of increased emissions from power plants due to altered air conditioning demand and from direct effects on atmospheric chemistry.

**What did the researchers do and find?:**

- We used computer models to calculate the air pollution and health impacts of a warmer climate with and without greater use of air conditioning and subsequent increases in harmful emissions from power plants.
- We found that concentrations of fine particulate matter (PM_2.5_) and ozone (O_3_) increase in a warmer climate and that 3.8% of the total increase in PM_2.5_ and 6.7% of the total increase in ozone (O_3_) are attributable to extra air conditioning use.
- We calculated that climate change alone increases summer air-pollution-related premature mortality by about 13,000 deaths due to PM_2.5_ and 3,000 deaths due to O_3_ (consistent with other studies).
- Increased air conditioning, specifically, accounts for 654 future summer PM_2.5_-related deaths (approximately $6 billion cost—based on a value of statistical life calculated from 26 studies—and a 4.8% increase above climate change impacts alone) and 315 O_3_-related deaths (approximately $3 billion cost and an 8.7% increase above climate change impacts alone).

**What do these findings mean?:**

- This is the first study to our knowledge to examine future air-pollution-related health damages of power plant emissions driven by increased electricity demand for air conditioning, a primary adaptation to warmer temperatures.
- Quantifying the extent to which air conditioning adaptation exacerbates climate-change-related air quality can improve decision-making, especially in both the health and electric power sectors.
- This analysis highlights the need for cleaner energy sources, energy efficiency, and energy conservation to meet our growing dependence on buildings’ cooling systems, while simultaneously mitigating the extent of climate change.

## Introduction

Climate change poses many health risks, from elevated risk of heat stroke to the broadening reach of vector-borne disease, food insecurity, and air pollution [1]. According to the Lancet Countdown on health and climate change, climate change “is affecting the health of populations around the world, today” [2]. Climate change has direct impacts on health and well-being from exacerbated extreme weather, extremes of the hydrologic cycle, and heat waves, as well as indirect effects such as increases in the burden of infectious disease, sea-level rise, ocean acidification, and climate-induced population displacement or conflict. Ultimately, these changes threaten access to clean air, water, and food, while potentially creating new health disparities and exacerbating existing ones. However, climate mitigation and adaptation strategies have the potential to address these issues and improve public health broadly. This study focuses on ambient air pollution, and the potential increase in adverse air-pollution-related health impacts associated with building air conditioning use, in response to warmer temperatures, highlighting the need for clean energy solutions as tools for improving public health.

Relationships between meteorological conditions and air quality have been established in past literature. For example, warmer temperatures and sunlight enhance production of biogenic, or natural, volatile organic carbons (VOCs) from certain plant species, which are precursors to both ozone and fine particulate matter [3,4]. Warm temperatures and sunlight also enhance ozone-forming reactions [5,6]. Pollutant concentrations decrease with increased air mixing [7,8] and precipitation [9,10], while increased humidity can increase formation of particulate matter [7,9]. Additional work has explored the impact of a warming climate on wildfire emissions [11–14], soil emissions of nitrogen oxides (NO_X_) [15], and NO_X_ from lightning [16–18]. Using these relationships, a number of studies have investigated the potential impact of climate change on air quality, particularly the response of ozone and particulate matter concentrations to warming temperatures [7,19–21]. Past studies assessing climate change impacts on air pollution often focused on the impact of climate change and meteorological variables (as well as biogenic, natural emissions) [7,19–21], the impact of future anthropogenic emission scenarios [22], or the combined impact of climate change and anthropogenic emission scenarios [10,22–27].

Air conditioning in buildings is a form of adaptation to warmer temperatures that could increase population health risks, by increasing power plant emissions on hot days. As air conditioning use increases to cool buildings, the increased demand for electricity is supplied by a mix of generation sources including fossil fuels, thus increasing harmful emissions. In this work, we deploy a novel interdisciplinary modeling effort to quantify the air pollution and health impacts of this climate change adaptation mechanism.

Few studies have explored the impact of climate change on health-damaging emissions from electricity generating units (EGUs), specifically emissions of nitrogen dioxide (NO_2_) and sulfur dioxide (SO_2_), but we know there is a relationship between power plant emissions and temperature through electricity demand in buildings. Buildings are the largest source of US electricity demand, responsible for more than 60% of demand in most states in the eastern US (https://www.eia.gov/electricity/data/state/). Electricity for cooling is a large component of this demand, with direct correlation to rising temperatures. Abel et al. showed that historical eastern US EGU emissions of NO_X_, SO_2_, and carbon dioxide (CO_2_) increase 3.3%–3.6% per 1 °C increase in daily temperature regionally over the summer [28], consistent with the findings of He et al. [29], who found an increase of 2.5%–4.0% per 1 °C increase in the eastern US states, and Dreschler et al. [30], who found an increase of 5.8% per 1 °C increase in California.

Additional emissions from increased air conditioning demand have been shown to have a significant impact on fine particulate matter (PM_2.5_), responsible for up to 87% of concentrations in the Pennsylvania–New Jersey–Maryland electricity grid interconnection during July 2006 heat wave conditions [31]. The hourly variability of EGU emissions due to temperature can increase PM_2.5_ mass, sulfate, and elemental carbon concentrations by 83%, 103%, and 310%, respectively, but the increase in emissions from anticipated heat-driven adaptation response is typically not included in air quality modeling studies [32]. Power plants have been extensively evaluated as a controllable source of pollution [33–35]. However, without action, residential and commercial buildings are expected to see an increase in cooling load and subsequent emissions [36,37]. Recent research has demonstrated the air-quality-related health benefit of the green building movement and reducing energy demand in buildings. MacNaughton et al. quantified the health benefits of US Leadership in Energy and Environmental Design (LEED)–certified buildings built from 2000 to 2016 as 172–405 avoided premature mortalities [38].

This is the first study to our knowledge to compare the impact of potential mid-century climate change on air quality with and without associated heat-driven changes in emissions from the electricity sector. This work advances the line of research characterizing health co-benefits from mitigation strategies [39–52] and the direct quantification of health damages from air pollution in a future climate [1,2,30,53–59]. This study builds upon a large body of epidemiological work relating air pollution and human health, including the studies utilized in the Environmental Protection Agency’s (EPA’s) Benefits Mapping and Analysis Program (BenMAP) [60].

## Methods

### Overview

We apply a system of linked numerical models to assess changes in building energy demand, electricity production, power sector emissions, air quality, and human health outcomes based on meteorology consistent with present-day conditions and a warm mid-century summer climate. We focus on the eastern US, where electricity production and use are connected through a regional power grid. This region also experiences levels of ground-level O_3_ and PM_2.5_ in exceedance of EPA health-based standards [61], and demographic trends in this area suggest continuing and increasing vulnerabilities to air pollution exposures [62–65].

**Fig 1:**
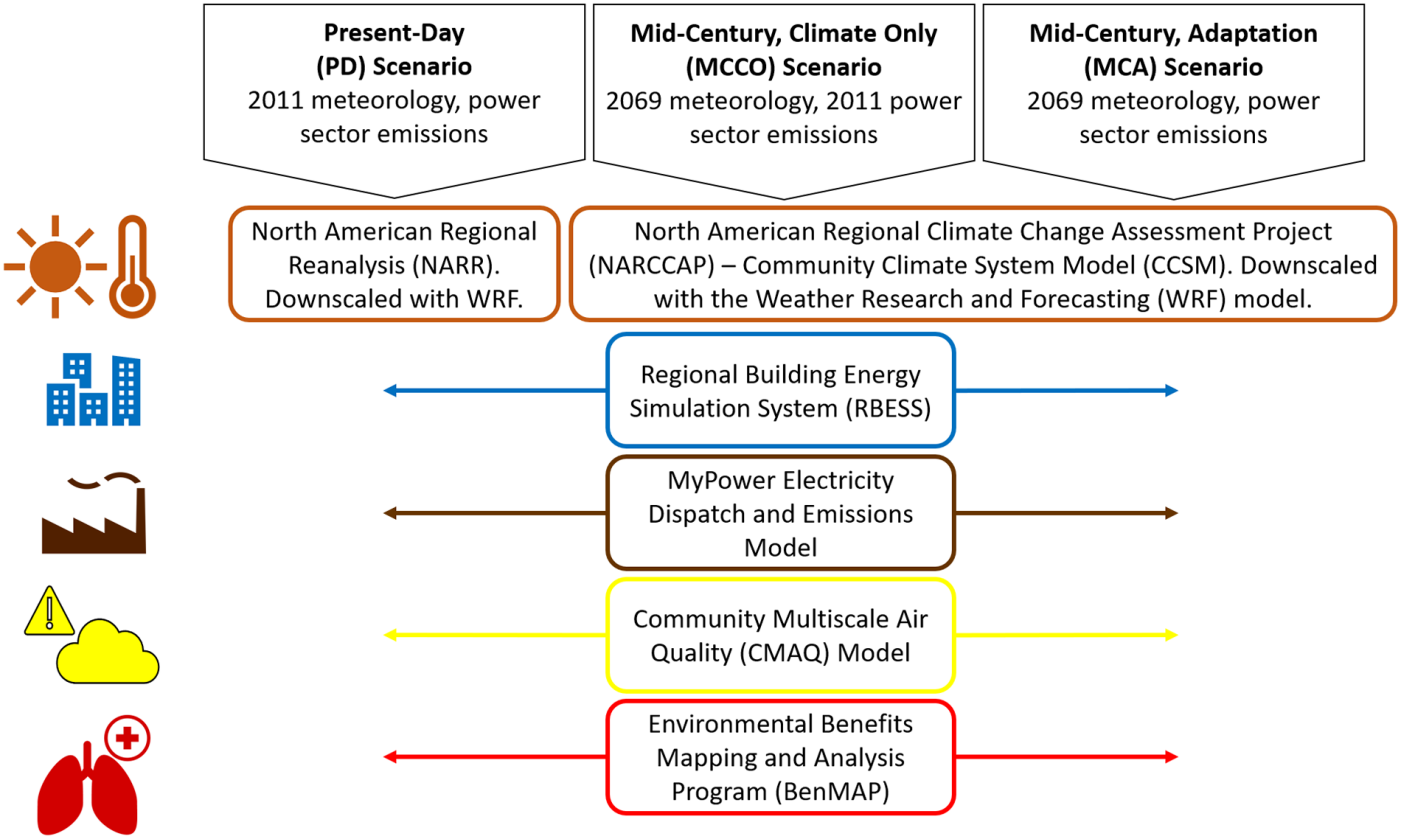
A visual representation of the methods used in this study.

Fig 1 provides a visual representation of the modeling system, which includes simulating present and future meteorology, electricity demand in buildings, electricity production and EGU emissions, air quality, and health impacts. For information on how to access the software used, please see S1 Text. We performed simulations for 3 scenarios using this linked model system, and a fourth simulation is used for validation of results, following standard practice for chemical transport modeling, for which uncertainty estimates are ill-suited and model evaluation is preferred (see S1 Text) [66–68]. Satellite-derived NO_2_ and previous studies are also used to validate results. Scenarios are shown below and outlined in Fig 1. Additionally, Table 1 shows the major data inputs for each model in the system.

**Table 1:** Major data inputs and sources for each step of the modeling framework.

Model	Input data needed	Input data source
**WRF**	Present-day meteorology	North American Regional Reanalysis (NARR): meteorological dataset (includes assimilated observations)
	Future meteorology	North American Regional Climate Change Assessment Program (NARCCAP) (Community Climate System Model [CCSM]): meteorological dataset selected from a suite of climate models
**RBESS**	Meteorology	WRF
	Representative building types	Built for this study based on Department of Energy DOE-2 platform
	Building stock	US Energy Information Administration (EIA) Commercial Buildings Energy Consumption Survey (CBECS), Residential Energy Consumption Survey (RECS), Manufacturing Energy Consumption Survey (MECS)
**MyPower**	Electricity demand	RBESS
	Power plants and characteristics	EPA’s National Electric Energy Data System (NEEDS), EPA’s Clean Air Markets Database
**CMAQ**	Power plant emissions	MyPower
	Other anthropogenic emissions	EPA’s National Emissions Inventory (NEI)
	Biogenic emissions	Model of Emissions of Gases and Aerosols from Nature (MEGAN)
	Meteorology	WRF
**BenMAP**	Population	US Census
	Baseline incidence	Many data sources; see [69]
	Concentration–response functions	Many studies; see health impact tables or BenMAP documentation for references [60,69]
	Air quality data	CMAQ

BenMAP, Environmental Benefits Mapping and Analysis Program; CMAQ, Community Multiscale Air Quality; EPA, Environmental Protection Agency; RBESS, Regional Building Energy Simulation System; WRF, Weather Research and Forecasting.

Three scenarios are simulated: the present-day climate, a mid-century climate with present-day emissions, and a mid-century climate with emissions from adaptation. Each of these scenarios utilizes meteorology from the WRF model for present-day and NARCCAP CCSM version 3 for mid-century. The RBESS is used to assess building energy demand, and MyPower is used to simulate electricity dispatch (production) and associated power sector emissions. CMAQ is used to simulate air quality, and BenMAP assesses the health outcomes from air quality changes.

#### Present-day (PD) scenario

This scenario represents present-day conditions for climate. Building energy demand and power sector (EGU) emissions are simulated for present-day conditions.

#### Mid-century climate-only (MCCO) scenario

This scenario represents warm mid-century conditions for climate, selected as described in detail below. Building energy demand and power sector (EGU) emissions remain constant as simulated for present-day conditions. This scenario represents the impact of climate change alone on air quality and health. There is no change in building activity or associated anthropogenic emissions from electricity demand.

#### Mid-century adaptation (MCA) scenario

This scenario represents warm mid-century conditions for climate. Building energy demand and power sector emissions are simulated using mid-century representative meteorology with inventory and performance held constant (modern natural gas power plants are assumed to provide the additional capacity needed to meet increased electricity demand). This scenario represents the impact of climate change and increased EGU emissions due to greater building air conditioning demand in response to warmer temperatures.

### Climate and meteorological modeling

Warm-climate simulations of air quality use meteorology downscaled from the NARCCAP [70] archive per Harkey and Holloway [71]. NARCCAP is a suite of climate data from several Global Climate Model–Regional Climate Model pairs built on the A2 emissions scenario of the Intergovernmental Panel on Climate Change (IPCC), a trajectory that most closely mirrors current global greenhouse gas emissions trends [72,73]. This emissions scenario assumes that little or no action is taken to mitigate climate change, which makes it appropriate for the goals of this study, to isolate the potential impact of increased power sector emissions on air quality. Thus, any successful future action to mitigate climate change would alleviate some of the damages calculated here.

Due to the computationally demanding simulations of this study, it is not feasible to consider a 30-year subset of mid-century years, as is recommended for climate impact studies. Rather, July of a single year from a single model in NARCCAP was selected to represent a warm, realistic mid-century summer, as discussed in detail in Harkey and Holloway [71]. We selected the year 2069 from CCSM version 3 [74], downscaled with the WRF model in NARCCAP, and further downscaled with WRF for this study to our 12 km by 12 km study domain. This year was chosen as the warmest year from the mean model in the suite, as shown in S1 Fig, adapted from Meier et al. [36]. To isolate the impact of climate change on air quality, we used the same 2011 emissions data and lateral boundary conditions for all simulations. Climate processes are considered to affect biogenic emissions, power plant emissions, and the transport of point-source anthropogenic emissions.

Present-day meteorology is downscaled in WRF from NARR for 2011 conditions, as described in Harkey and Holloway [71]. The NARR model assimilates measured meteorological data to produce a gridded, continuous dataset [75]. We focus on July 2011 as representative of peak summertime electricity demand and the summer high O_3_ season, and consistent with the latest NEI at the time of modeling.

Therefore, findings are separated into the impacts of meteorology representative of 2 summer climate scenarios, the present-day climate and a warm mid-century climate representative of climate change mitigation inaction (July 2011 and July 2069). Meteorological conditions for July in the warm mid-century climate used in the MCCO and MCA scenarios are on average approximately 3.5 °C (29.1 °C versus 25.6 °C, 13.7%) warmer in the eastern US region than in the present-day.

### Building energy demand modeling

Present-day and warm mid-century meteorology were input to the RBESS, a modeling process developed following Schuetter et al. [76] and used here to determine the response of building energy demand to meteorology. This process merges industry-standard building energy modeling techniques using the DOE-2 software (developed by James J. Hirsch & Associates and Lawrence Berkeley National Laboratory) and regional building stock data with the meteorology discussed above following Meier et al. [36], which describes the methodology used here in detail. Building stock data were provided by the US EIA through the Commercial Buildings Energy Consumption Survey (CBECS), the Manufacturing Energy Consumption Survey (MECS), and the Residential Energy Consumption Survey (RECS). The building stock was held static under both the present-day and warm-climate scenarios. The simulation was calibrated using historical 2007 electricity data from a US EPA compilation of Federal Energy Regulatory Commission (FERC) data. Use of the present-day building stock was not meant to be predictive but was chosen to bound the potential damages of climate inaction.

### Electricity sector dispatch modeling

Building energy demand was input to the MyPower model, a load duration curve (LDC) electricity dispatch model, used to simulate plant-level electricity production and emissions of NO_X_, SO_2_, and CO_2_. Detailed methodology for MyPower is described in Meier et al. [36]. Data for power plant characteristics including heat rates and emissions rates were derived from NEEDS, a part of the US EPA’s Power Sector Modeling Platform, and modified to reflect data reported in the US EPA’s Clean Air Markets Database through 2013. Present-day conditions reflect electricity sector characteristics through 2011. Warm-climate conditions reflect planned changes to the electricity grid. Existing renewable energy portfolio standards are met through a combination of technologies reported in the Database of State Incentives for Renewables & Efficiency (DSIRE) database [77]. Nuclear power plants are retired as specified by existing operating licenses, and applications for new constructions are as reported by the Nuclear Regulatory Commission [78]. In the warm-climate scenarios, power plants are assumed to maintain “resource adequacy” such that generating capacity exceeds the highest single hour of demand by 15%. The additional required power is supplied through new construction of natural gas power plants (70% combined-cycle, 30% single-cycle) with characteristics based on the Annual Energy Outlook from the US EIA [79]. All existing plants not retired are not modified.

Scenario selection, specifically using the present-day building stock and power plants, is not meant to be predictive, but to quantify the portion of future damages that could be alleviated by changes to the building sector and electricity sector. The scenario was chosen to describe the potential damages of interactions between climate, energy use, and air quality through this previously unstudied mechanism.

### Air quality modeling

Air quality simulations were performed using the CMAQ model version 5.0.1 [67,80]. Anthropogenic emissions were input from the EPA 2011 NEI [81], and biogenic emissions were simulated using MEGAN version 2.1 [82]. We focus on July 2011 conditions for the present-day as representative of peak summertime electricity demand and production within the high O_3_ season, consistent with past literature and the latest available NEI emissions data at the time of modeling [31]. S1 Text includes validation of results and discussion of model performance.

EGU emissions from MyPower were gridded for use in CMAQ and substituted for NO_X_ and SO_2_ emissions in the NEI. Emissions of NO_X_ were assigned constant partitioning of 85% NO and 15% NO_2_. Chemical species that are contained in the NEI but not directly calculated by MyPower are listed in S1 Table, with associated discussion.

All CMAQ simulations were configured with “AERO6” aerosol chemistry [67], in-line photolysis, and the Carbon Bond 5 (CB05) chemical mechanism with updated toluene and chlorine chemistry [83,84]. Simulations do not include estimates of emissions from fires but do include in-line estimates of lightning-generated NO_X_. CMAQ was run with 25 vertical layers, a 12 km by 12 km horizontal resolution over the eastern US, and boundary conditions taken from a month-averaged run of present-day conditions with NEI emissions estimates over the continental US, which in turn used boundary conditions from the Model for Ozone and Related Chemical Tracers, version 4 [85].

We chose to run simulations through CMAQ for only July as these simulations were the most computationally expensive part of our linked model system. As results represent estimates based on only a single year of climate simulations, findings are meant as exploratory and illustrative, and as such the marginal limitation of extrapolating July results as representative of summer (and summer as representative of annual impacts) is small. Future research could utilize less computationally expensive methods to run more scenarios over longer and more representative timescales, but the complex mechanisms included in CMAQ are necessary to explore the impacts of power sector emissions on air quality in a changing climate through the new relationship described here. We also chose July based on 3 additional simulations that were run (baseline, baseline with fires, mid-century baseline) and 2 others that were prepared but not run (present and future emissions approximated through temperature versus emissions relationships defined as in Abel et al. [28]). These additional simulations influenced the decision to simulate July only, but did not contribute to the objectives of this paper and were therefore disregarded.

### Health impacts assessment

We assessed increased incidence of premature mortality and morbidity associated with exposure to higher daily mean PM_2.5_, maximum daily 8-hour average (MDA8) O_3_, and maximum daily 1-hour O_3_ using the EPA’s BenMAP–Community Edition version 1.3 [60]. BenMAP calculates the incidence of adverse health outcomes given a change in air quality. Expert-derived PM_2.5_ exposure–response (or concentration–response [C-R]) functions and pooling methods used for the US EPA 2012 Regulatory Impact Analysis and O_3_ C-R functions used for the 2008 National Ambient Air Quality Standards (NAAQS) evaluations are applied in this analysis [86–88]. These standard EPA configurations are available with the BenMAP software. Population is held constant for 2011 in all scenarios. Comparative analysis of the benefits of air conditioning in buildings for reducing direct heat-related mortality versus air pollution effects from air-conditioning-related electricity demand is beyond the scope of this study.

BenMAP combines population data from the US Census, baseline health outcome incidence data provided from several sources but primarily the Centers for Disease Control and Prevention (CDC) (outlined in Appendix D of [69]), and an effect estimate from the chosen C-R function with specified changes in gridded air quality data to quantify health impacts. Each exposure–response function and pooling of incidence and valuation was run in a 5,000-member Monte Carlo ensemble to calculate mean impacts and associated uncertainty. Pooling methods are used to combine results for similar health endpoints across C-R functions as an alternative to meta-analysis. The techniques used here follow standard EPA methods including user-assigned weighting, random effects, fixed effects, addition, and subtraction to combine results of studies as described in Appendix K of [69]. Here we focus on mortality, which is not pooled as standard practice in the EPA methodology. Amongst mortality results, the American Cancer Society’s Cancer Prevention Study II, used for PM_2.5_-related mortality estimates, is especially relevant because the study data include the most representative exposure sites in the US and a follow-up period of 18 years [89]. Health impacts based on maximum daily 1-hour O_3_ are simulated but not pooled, as there is no standard EPA methodology based on maximum daily 1-hour metrics, and these results are used primarily for comparison. Valuation to monetize the costs of exacerbated air pollution is performed according to standard EPA configurations by assigning a value to each health effect through a combination of willingness to pay and cost of illness (e.g., value of a statistical life) methods, then applying that to calculated incidences [60]. All costs are presented in US dollars.

Impact estimates are based on exacerbated pollution in July alone. Annual impacts are calculated as a 3-month summer average based on July modeling. Thus, we take July as representative of the entire summer and triple our calculated results to arrive at a summer estimate. This is a reasonable assumption for the changes in air pollution and health impacts analyzed here, especially given the focus on the incremental impact of adaptation. Values presented in tables are for July exposure alone and have not been tripled. Average baseline scenario concentrations of PM_2.5_ and O_3_ from July modeling are applied outside of July in all calculations to isolate changes. Summer results are a good estimate for annual impacts although they are likely conservative as we would also expect spring and fall to exhibit some increased air pollution and adverse health outcomes. Winter air quality conditions are less influenced by the electricity sector. Impacts for estimated annual/summer exposure are provided in the text while July exposure impacts are presented in the tables.

All health impact functions for PM_2.5_-related mortality apply an annual average air pollution metric, calculated from daily mean values with changes only in July. The daily mean is used directly for many morbidity functions. All impacts calculated by BenMAP at any timescale are summed and reported annually by the model as standard practice. Therefore, values provided in the tables are annual impacts based on July exposure, while values provided in the text are annual impacts based on estimated annual exposure calculated as a 3-month summer average based on July modeling, as discussed above. O_3_-related premature mortality functions are based on metrics of MDA8 or maximum daily 1-hour O_3_, with Jerrett et al. [90] the only study based on an annual average metric. Justification for modeling only July is discussed in detail above but centers on balancing computational demands with the exploratory and representative (rather than predictive) nature of this study. We present in the main text primarily the results for mortality, which by standard methods are not pooled. Please see S2–S4 Tables for morbidity results.

## Results

### Emissions and air quality

**Fig 2:**
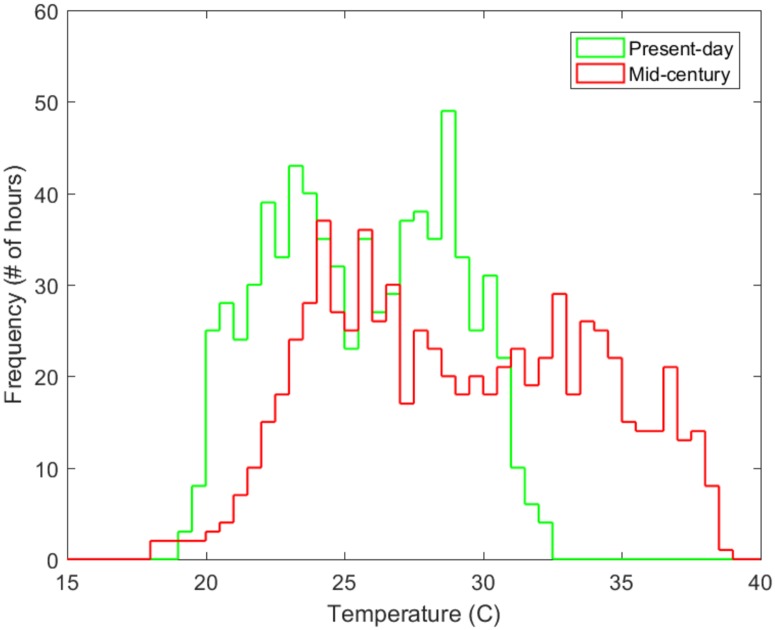
A histogram of regional average hourly temperatures. A histogram of regional average hourly temperatures is presented for July in the present-day and in the warm mid-century climate. Present-day mean: 25.6 °C; minimum: 19.1 °C; maximum: 32.4 °C. Mid-century mean: 29.1 °C; minimum: 18.3 °C; maximum: 38.5 °C.

Changes in energy demand associated with warmer temperatures are driven by the distribution of temperatures at hourly or even sub-hourly scales. Fig 2 shows a histogram of regional (eastern US) average hourly temperatures over the month of July for current and mid-century conditions. Results show a shift in the maximum ambient temperature from 32.4 °C (present) to 38.5 °C (future), an 18.8% increase. The mid-century scenario exhibits a decrease in the frequency of colder temperatures and an increase in the frequency of warmer temperatures.

**Fig 3:**
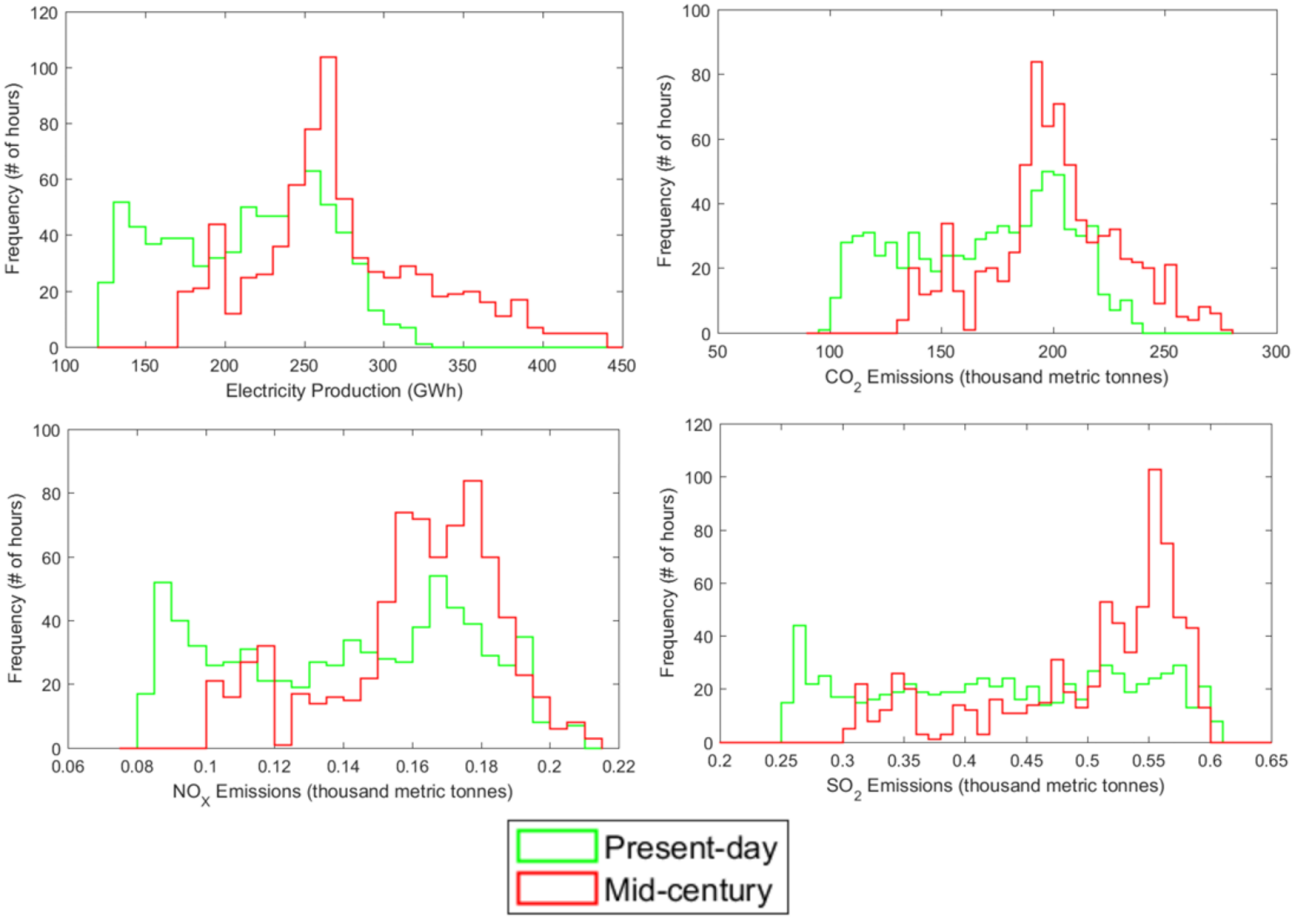
Histograms of hourly electricity production and emissions. Histograms are provided for regionally summed hourly electricity production, CO_2_ emissions, nitrogen oxide (NO_X_) emissions, and SO_2_ emissions for July in the present-day and warm mid-century warm climate scenarios. For electricity production: present-day mean: 212.9 GWh; minimum: 120.4; maximum: 320.3. Mid-century mean: 274.2 GWh; minimum: 172.0; maximum: 438.0. For CO_2_ emissions: present-day mean: 168,800 tonnes; minimum: 99,800; maximum: 238,800. Mid-century mean: 200,100 tonnes; minimum: 132,700; maximum: 276,500. For NO_X_ emissions: present-day mean: 140 tonnes; minimum: 80; maximum: 210. Mid-century mean: 160 tonnes; minimum: 100; maximum: 210. For SO_2_ emissions: present-day mean: 430 tonnes; minimum: 250; maximum: 610. Mid-century mean: 500 tonnes; minimum: 300; maximum: 590.

The higher temperatures seen in the mid-century scenarios drive changes in electricity demand, production, and associated emissions. Fig 3 shows the hourly distribution of electricity production and emissions for current and future climates. These results show the response of electricity production to ambient temperature through demand for air conditioning. Under the future climate assumptions, regionally summed average hourly electricity demand increases from 213 to 274 GWh (28.6%), and regionally summed average hourly eastern US CO_2_ emissions increase from 169,000 to 200,000 metric tonnes (18.3%). Thus, adaptation through air conditioning use also constitutes a positive climate feedback.

The change in maximum CO_2_ is not as large as the change in electricity production because additional capacity in mid-century (necessary to meet increased demand) is generated by natural gas power plants based on the US EIA’s Annual Energy Outlook, which emit less carbon than the current mix of generation sources [36]. We find that electricity production and emissions in the present-day exhibit a more uniform distribution than does temperature (Fig 2). This difference is due to the changing sensitivity of electricity generation as a function of temperature, with responsiveness increasing at higher temperatures and decreasing at cooler temperatures, when building cooling is less important. The distribution becomes less uniform in the mid-century climate as temperature dependence plays a greater role compared to other end uses of electricity.

Trends in the distribution of hourly electricity production and CO_2_ emissions more closely follow changes in temperature than do emissions of NO_X_ and SO_2_, as shown in Fig 3. Overall, emissions in the future climate scenario increase 13.7% for NO_X_ and 17.2% for SO_2_, but the maximum hourly emissions rate does not increase for either NO_X_ or SO_2_. Rather, the increase in average hourly emissions of NO_X_ and SO_2_ occurs from greater frequency of emissions on the higher end of the present-day emissions distribution. Even as electricity demand increases, new peak electricity demand in the model is met by natural gas power plants that have little impact on NO_X_ and SO_2_ emissions during peak conditions. Simulating likely retirements of coal-fired power plants and market-driven renewable energy investments would also result in lower emissions than found here, where we maintain the existing power plant inventory to explore the arising interactions between climate, energy production, and air quality without being predictive. This highlights the importance of considering cleaner energy sources in reducing future harmful emissions.

Overall, a 3.5 °C warmer summer is responsible for an increase in hourly average building energy demand of 28.6%. The air conditioning adaptation response to climate change in the eastern US is thus responsible for hourly average emissions increases of 13.7% for NO_X_, 17.2% for SO_2_, and 18.5% for CO_2_.

We analyzed air quality in the PD (present-day climate, present-day EGU emissions), MCCO (mid-century climate only), and MCA (mid-century adaptation) scenarios as described in the Methods. On a regional average, we find that climate change alone (MCCO versus PD) increases PM_2.5_ by 58.6% (2.50 μg/m^3^) and O_3_ by 14.9% (8.06 parts per billion by volume [ppbv]). A larger change is found when comparing the present day to the mid-century adaptation scenario, which includes building air conditioning (MCA versus PD). In that case, PM_2.5_ increases 61.1% (2.60 μg/m^3^) and O_3_ increases 15.9% (8.64 ppbv). Overall, 2.5% of the 61.1% increase in PM_2.5_ and 1.0% of the 15.9% increase in O_3_ are attributable to adaptive behavior (extra air conditioning use).

**Fig 4:**
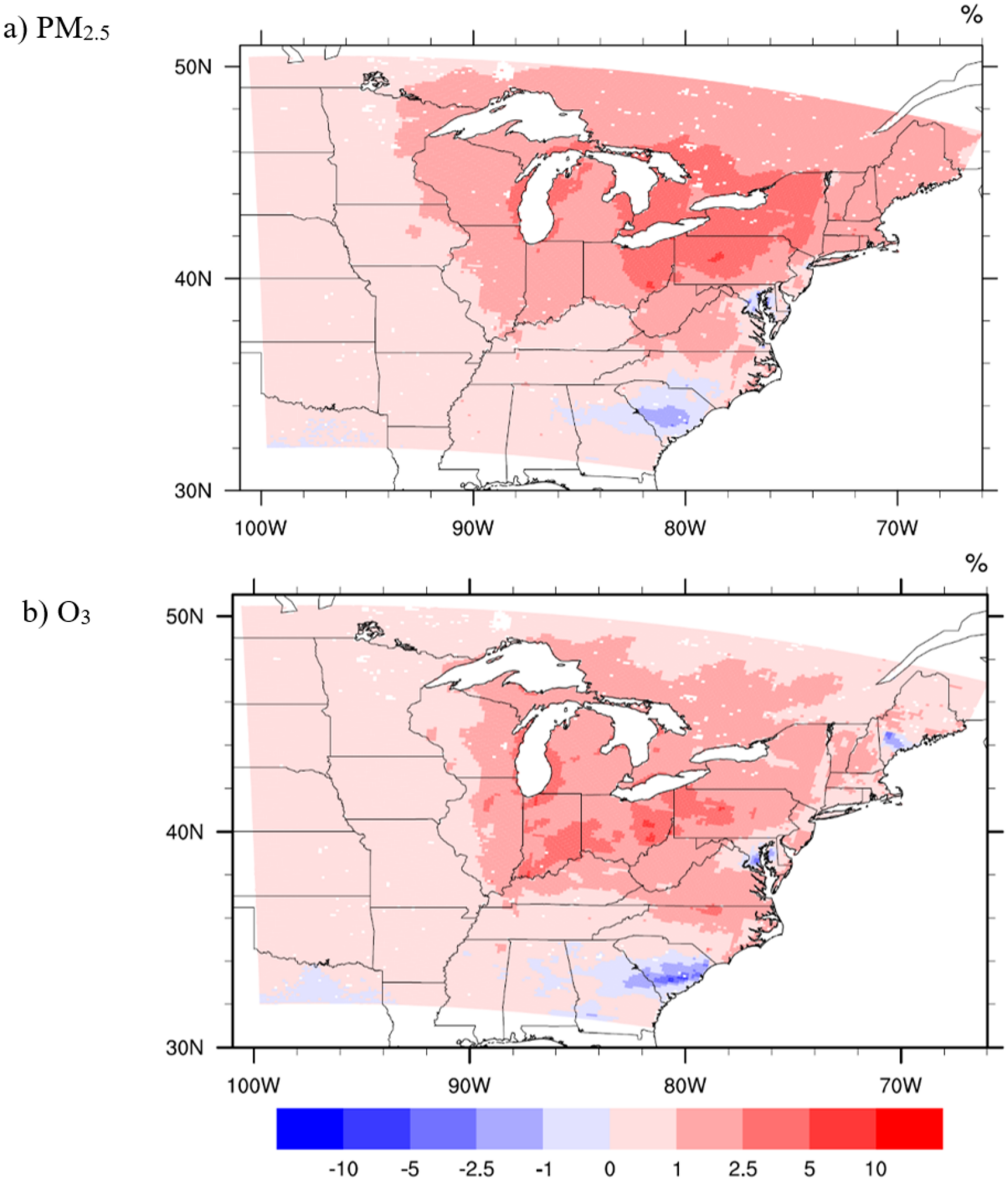
Change in ambient air pollution concentrations. Maps of the percentage change in (a) PM_2.5_ and (b) O_3_ from the warm mid-century climate-only (MCCO) scenario to the warm mid-century adaptation (MCA) scenario. Red shows concentrations that are greater in the MCA scenario compared to MCCO, while blue shows a decrease in concentrations compared to MCCO. Axes show latitude and longitude.

**Fig 5:**
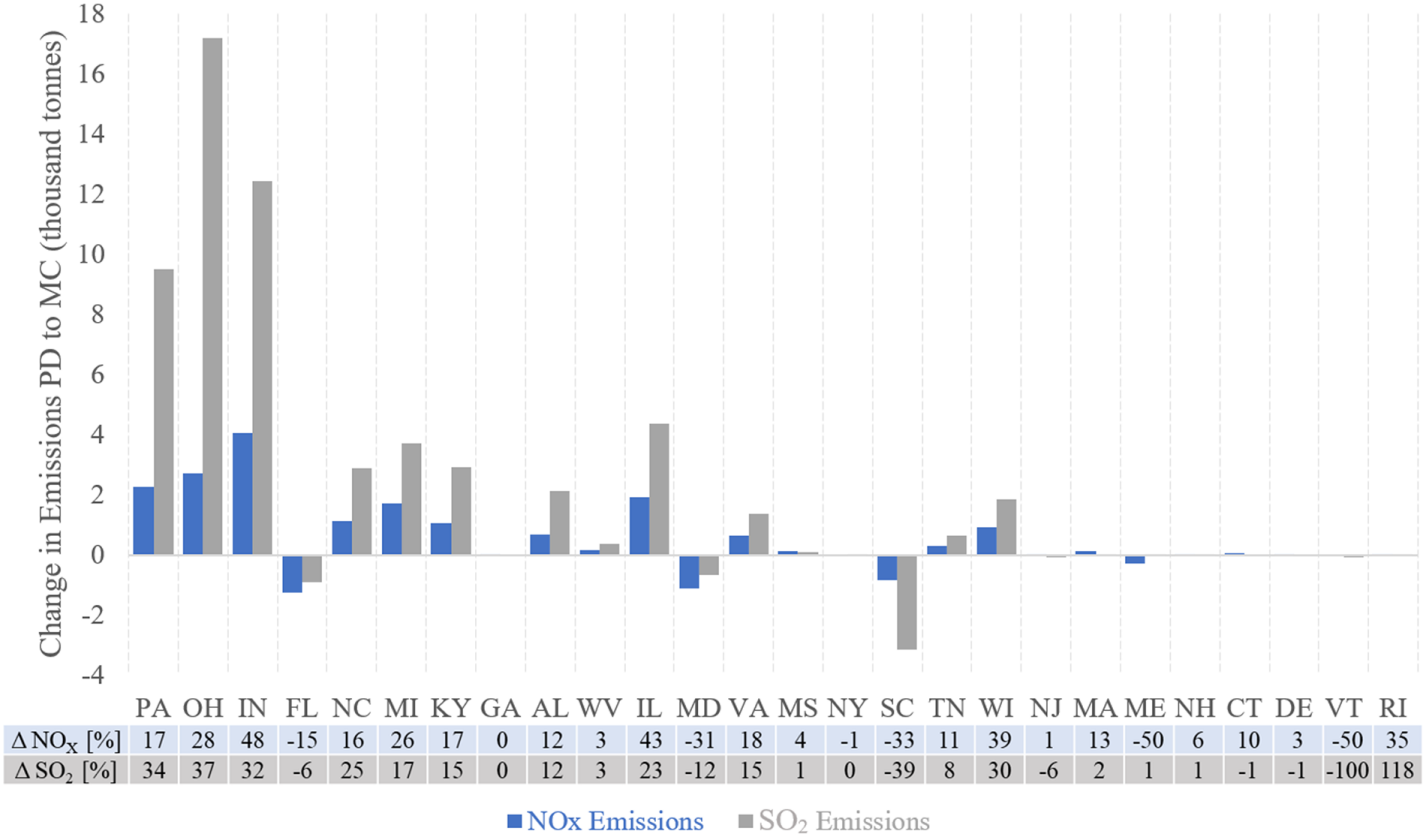
Change in emissions by state. The state by state changes in nitrogen oxide (NO_X_) and SO_2_ emissions from the present-day (PD) to mid-century (MC) as an absolute value (designated by the bars) and as a percentage (as listed).

The July average change in each pollutant due to building energy use is shown in Fig 4 for PM_2.5_ (Fig 4a) and MDA8 O_3_ (Fig 4b). Increases in PM_2.5_ from the MCCO to the MCA scenario (Fig 4a) are highest (as high as >5%) in and downwind of the Ohio River Valley, coincident with the highest concentration of fossil fuel, especially coal-fired, power plants and the greatest increase in EGU emissions. A small decrease (<2.5%) in concentrations is observed in the southeast, centered over South Carolina and the Chesapeake Bay. This is primarily due to a decrease in emissions in these regions (as seen in Fig 5) associated with power plant dispatch changes (see Meier et al. [36]).

**Fig 6:**
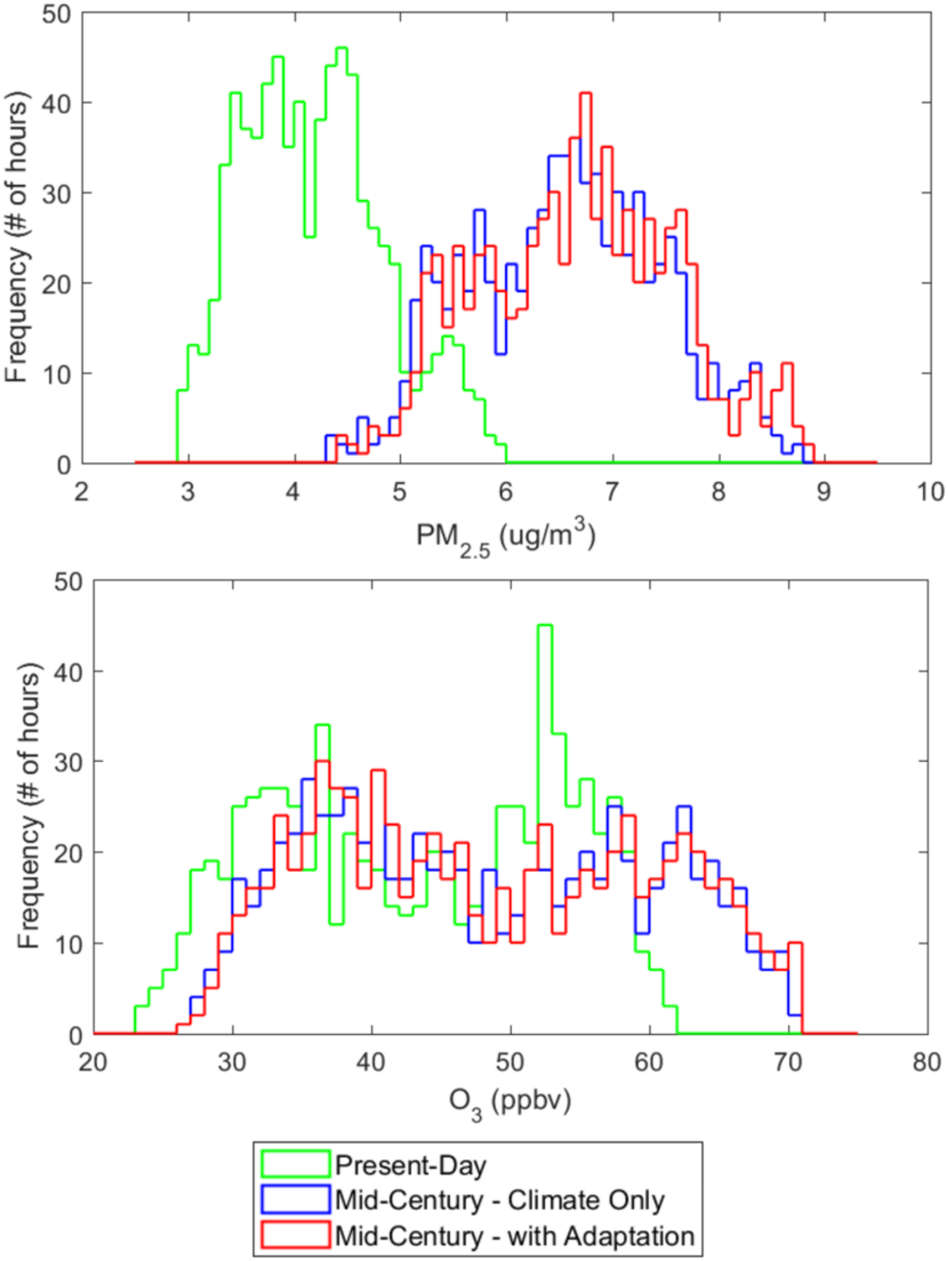
Histograms of ambient air pollutant concentrations. Histograms of regional average hourly concentrations of PM_2.5_ (μg/m^3^) and O_3_ (parts per billion by volume [ppbv]) for July in the present-day (PD) scenario, the warm mid-century climate-only (MCCO) scenario, and the warm mid-century adaptation (MCA) scenario. For PM_2.5_ concentrations: PD mean: 4.19 μg/m^3^; minimum: 2.91; maximum: 5.98. MCCO mean: 6.57 μg/m^3^; minimum: 4.37; maximum: 8.75. MCA mean: 6.67 μg/m^3^; minimum: 4.48; maximum: 8.87. For O_3_ concentrations: PD mean: 43.4 ppbv; minimum: 23.6; maximum: 61.7. MCCO mean: 48.0 ppbv; minimum: 26.4; maximum: 70.2. MCA mean: 48.4 ppbv; minimum: 26.6; maximum: 70.9.

We examined the distribution of regional average concentrations as a function of air pollution level in Fig 6. The number of hours with pollution at the highest levels increases due to climate change alone, and further rises given greater emissions of NO_X_ and SO_2_ associated with higher climate-induced electricity demand. For PM_2.5_, the minimum regional average concentration simulated under a future climate (4.37 μg/m^3^ for MCCO) is above the average value for present-day (4.26 μg/m^3^). Present-day values range from a minimum of 2.91 μg/m^3^ to a maximum 5.98 μg/m^3^. The highest regional average concentrations modeled under a future climate (8.75 μg/m^3^ for MCCO) are higher than we see at any time in the present-day simulation. The additional consideration of adaptation through air conditioning use further increases the minimum and maximum values to 4.48 μg/m^3^ and 8.87 μg/m^3^, respectively.

Biogenic emissions, enhanced under a warmer climate, are the dominant contributor to the MCCO increase in PM_2.5_. This impact is sensitive to the choice of chemical mechanism in the atmospheric model and details regarding the formation of secondary organic aerosol as a function of volatile organic compounds. Past studies have suggested that the CB05 mechanism in CMAQ may have errors in the representation of this atmospheric chemical process [91–93]. Thus, while the direct impact of climate on PM_2.5_ is notable, we focus our discussion on the changes due to building energy use (i.e., MCCO versus MCA).

Modeled EGU emissions of SO_2_ increase by 17.2%, and NO_X_ by 13.7%, due to building energy use in the future climate (state-by-state variation shown in Fig 5). This increase in EGU emissions results in increases in sulfate particulate matter (SO_4_^2−^, 5.8% as compared to MCCO, or 0.09 μg/m^3^) and nitrate PM (NO_3_^−^, 3.1% as compared to MCCO, or 0.7 × 10^−3^ μg/m^3^).

Ozone exhibits many of the same patterns as exhibited by PM_2.5_. However, the increase in hourly O_3_ is not as pronounced from the present-day to mid-century scenarios as seen for PM_2.5_. In the case of O_3_, adaptive behavior is responsible for an approximately 1% increase in O_3_. Like PM_2.5_, O_3_ increases across most of the region (Fig 4), with the greatest increases in and downwind of the Ohio River Valley (as high as >5%) due to increases in EGU NO_X_ emissions. Small decreases due to localized emissions decreases from changes in electricity dispatch are also evident over South Carolina and Chesapeake Bay, as well as a highly localized decrease in Maine and a very small decrease along the Texas domain boundary.

Building energy use also results in a greater frequency of high O_3_ days (Fig 6). Note that the highest regional (eastern US) average hourly concentrations exceed the current NAAQS for MDA8 O_3_ of 70 ppbv [87]. These metrics are not directly comparable as standards are met or achieved at the county or state level and are based on the fourth highest annual MDA8, whereas we present regional average hourly concentrations. Additionally, the standards may be lowered by mid-century, but this comparison highlights the relevance of results to attainment of regulatory standards. Overall, adaptation causes a 4.5% increase in the number of high O_3_ hours (defined as when regional average hourly O_3_ exceeds 60 ppbv) and a 22% increase in the number of high PM_2.5_ hours (defined as when regional average hourly PM_2.5_ exceeds 8 μg/m^3^). Note that the NAAQS for PM_2.5_ is an annual average concentration of 12 μg/m^3^. However, our analysis is limited to a sample size of the 744 hours of July and not directly comparable to the NAAQS.

### Health impacts

Increased exposure to PM_2.5_ and O_3_ increases risk of premature mortality, which we quantify using BenMAP. Health impact functions are based on EPA-selected epidemiological studies and expert elicitation used in the US EPA 2012 Regulatory Impact Analysis for revisions to the NAAQS for particulate matter. Tables 2 and 3 summarize the changes to premature mortality from increased July exposure to PM_2.5_ and O_3_ under each scenario (negative numbers indicate adverse health outcomes and monetary costs). Morbidity impacts are summarized in S2–S4 Tables.

**Table 2:** PM_2.5_-related mortality results summed regionally for July exposure and displayed for each scenario comparison.

PM_2.5_ (24-hour mean) C-R function source	MCA–MCCO		MCCO–PD		MCA–PD	
	Mortality incidence (95% CI)	Valuation (95% CI) [billions of dollars]	Mortality incidence (95% CI)	Valuation (95% CI) [billions of dollars]	Mortality incidence (95% CI)	Valuation (95% CI) [billions of dollars]
Expert A	−319	−3	−6,459	−60	−6,779	−63
	(−657, −37)	(−10, 0)	(−13,348, −749)	(−202, −3)	(−14,010, −786)	(−212, −3)
Expert B	−261	−2	−4,956	−46	−5,201	−48
	(−561, −17)	(−9, 0)	(−10,980, −115)	(−184, −1)	(−11,522, −122)	(−193, −1)
Expert C	−251	−2	−5,073	−47	−5,325	−49
	(−446, −61)	(−7, 0)	(−9,037, −1,223)	(−146, −3)	(−9,486, −1,284)	(−153, −4)
Expert D	−176	−2	−3,565	−33	−3,742	−35
	(−302, 0)	(−5, 0)	(−6,117, 0)	(−104, 0)	(−6,420, 0)	(−109, 0)
Expert E	−415	−4	−8,409	−78	−8,827	−82
	(−658, −149)	(−11, 0)	(−13,373, −3,020)	(−229, −6)	(−14,037, −3,169)	(−240, −7)
Expert F	−239	−2	−4,281	−40	−4,497	−42
	(−353, −107)	(−6, 0)	(−6,660, −2,027)	(−114, −3)	(−6,992, −2,128)	(−120, −4)
Expert G	−147	−1	−2,966	−27	−3,113	−29
	(−278, 0)	(−5, 0)	(−5,622, 0)	(−100, 0)	(−5,901, 0)	(−105, 0)
Expert H	−183	−2	−3,707	−34	−3,891	−36
	(−521, 0)	(−7, 0)	(−10,562, 0)	(−142, 0)	(−11,086, 0)	(−149, 0)
Expert I	−248	−2	−5,028	−46	−5,277	−49
	(−442, 0)	(−7, 0)	(−8,954, 0)	(−149, 0)	(−9,398, 0)	(−156, 0)
Expert J	−202	−2	−4,085	−38	−4,288	−40
	(−468, −16)	(−7, 0)	(−9,485, −314)	(−136, −2)	(−9,956, −330)	(−143, −2)
Expert K	−29	0	−418	−4	−440	−4
	(−135, 0)	(−2, 0)	(−2,394, 14)	(−29, 0)	(−2,513, 13)	(−31, 0)
Expert L	−183	−2	−3,121	−29	−3,276	−30
	(−433, −1)	(−6, 0)	(−8,218, −2)	(−119, 0)	(−8,625, −2)	(−125, 0)
Krewski et al. [89]	−122	−1	−2,476	−23	−2,599	−24
	(−162, −83)	(−3, 0)	(−3,279, −1,673)	(−62, −2)	(−3,442, −1,755)	(−65, −2)
Lepeule et al. [94]*	−280	−3	−5,682	−52	−5,962	−55
	(−420, −140)	(−7, 0)	(−8,537, −2,828)	(−150, −5)	(−8,959, −2,968)	(−157, −5)

Expert functions were used for the Environmental Protection Agency 2012 Regulatory Impact Analysis [69,88]. Elicitation was performed to help characterize uncertainty of PM_2.5_-related mortality estimates.

*Lepeule et al. [94] is based on an age range of 25–99 years while all others are based on an age range of 30–99 years.

MCCO, mid-century climate-only; MCA, mid-century adaptation; PD, present-day; C-R, concentration–response.

**Table 3:** O_3_-related mortality results summed regionally for July exposure and displayed for each scenario comparison.

O_3_		MCA–MCCO		MCCO–PD		MCA–PD	
Health outcome	C-R function source	Incidence (95% CI)	Valuation (95% CI) [millions of dollars]	Incidence (95% CI)	Valuation (95% CI) [millions of dollars]	Incidence (95% CI)	Valuation (95% CI) [millions of dollars]
Mortality all cause	Bell et al. [96]	−103	−955	−1,057	−9,760	−1,149	−10,600
		(−158, −49)	(−2,752, −84)	(−1,634, −493)	(−28,323, −848)	(−1,775, −536)	(−30,775, −922)
Mortality all cause	Levy et al. [97]	−146	−1,350	−1,509	−14,000	−1,640	−15,200
		(−192, −100)	(−3,678, −126)	(−2,010, −1,019)	(−37,984, −1,297)	(−2,182, −1,107)	(−41,264, −1,410)
Mortality all cause	Zanobetti & Schwartz [98]	−66	−609	−667	−6,160	−725	−6,690
		(−97, −35)	(−1,730, −54)	(−984, −353)	(−17,474, −546)	(−1,069, −385)	(−18,999, −593)
Mortality cardiopulmonary	Huang et al. [95]	−38	−353	−407	−3,760	−440	−4,060
		(−62, −14)	(−1,045, −29)	(−672, −149)	(−11,203, −309)	(−727, −161)	(−12,111, −334)
Mortality non-accidental	Bell et al. [99]	−29	−270	−300	−2,770	−325	−3,000
		(−49, −10)	(−813, −22)	(−502, −99)	(−8,353, −223)	(−545, −107)	(−9,054, −242)
Mortality non-accidental	Ito et al. [100]	−132	−1,220	−1,398	−12,900	−1,513	−14,000
		(−184, −79)	(−3,392, −112)	(−1,992, −820)	(−36,202, −1,174)	(−2,155, −888)	(−39,190, −1,272)
Mortality non-accidental	Schwartz [101]	−44	−411	−458	−4,230	−496	−4,580
		(−75, −14)	(−1,248, −33)	(−780, −140)	(−12,914, −335)	(−845, −152)	(−13,996, −364)
Mortality non-accidental	Smith et al. [102]	−29	−266	−296	−2,730	−321	−2,960
		(−66, 8)	(−978, 63)	(−679, 79)	(−10,097, 640)	(−736, 86)	(−10,942, 695)
Mortality non-accidental	Smith et al. (2) [102]	−36	−333	−370	−3,420	−401	−3,710
		(−55, −18)	(−956, −30)	(−563, −179)	(−9,833, −304)	(−610, −194)	(−10,658, −330)
Mortality all cause	Levy et al. [97]^a^	−123	−886	−1,477	−10,600	−1,603	−11,507
		(−162, −85)	(−1,576, −308)	(−1,965, −998)	(−18,985, −3,653)	(−2,132, −1,084)	(−20,605, −3,966)
Mortality non-accidental	Ito & Thurston [103]^a^^,^^b^	−87	−623	−1,037	−7,440	−1,125	−8,075
		(−154, −19)	(−1,369, −125)	(−1,879, −224)	(−16,571, −1,455)	(−2,039, −243)	(−17,976, −1,580)
Mortality non-accidental	Ito et al. [100]^a^	−55	−395	−647	−4,645	−703	−5,044
		(−73, −37)	(−706, −136)	(−863, −434)	(−8,334, −1,598)	(−937, −471)	(−9,049, −1,735)
Mortality non-accidental	Schwartz [101]^a^	−47	−341	−557	−3,999	−605	−4,342
		(−80, −15)	(−721, −85)	(−950, −170)	(−8,507, −987)	(−1,032, −185)	(−9,236, −1,073)
Mortality respiratory	Jerrett et al. [90]^a^^,^^b^	−55	−397	−623	−4,473	−679	−4,872
		(−92, −19)	(−830, −104)	(−1,039, −209)	(−9,360, −1,165)	(−1,131, −228)	(−10,196, −1,269)

^a^These functions are based on maximum daily 1-hour O_3_ concentrations.

^b^These functions have age ranges other than 0–99 years: 30–99 years for Jerrett et al. [90] and 18–99 years for Ito and Thurston [103].

MCCO, mid-century climate-only; MCA, mid-century adaptation; PD, present-day; C-R, concentration–response.

As discussed in the Methods, we present annual impacts (estimated as a 3-month summer average based on July modeling) in the text, while results in tables are annual impacts based on changes to July exposure only. We include 14 C-R functions for PM_2.5_-related adult mortality, with each function reported separately. The change in mortality incidence and the economic valuation of this loss of life are shown in Table 2 with 95% confidence intervals based on the reported uncertainty underlying each relative risk point estimate simulated in 5,000-member Monte Carlo ensembles. Morbidity impacts are reported in S2 Table, and validation of air quality results is provided in S1 Text. For O_3_, we calculated mortality based on MDA8 concentrations as well as maximum daily 1-hour concentrations as shown in Table 3 (morbidity impacts are reported in S3 and S4 Tables).

For the impact of adaptation alone (MCA–MCCO), the 14 functions for PM_2.5_ exhibit a range of mean increases in mortality from 87 to 1,245 deaths ($0 to $12 billion in costs) annually and an average of 654 deaths ($6 billion); see Table 2 for individual study confidence intervals. The average 95% CI across studies is 131 to 1,251 deaths. Adapting to climate change as calculated here accounts for a 4.8% increase over the impacts from climate change alone (MCCO–PD), which on average causes 12,906 additional premature deaths (mean estimate range across studies: 1,254 to 25,227) with mean costs of $120 billion (mean estimate range across studies: $12 billion to $234 billion). The average 95% CI across studies is 2,558 to 24,978 deaths. The total impact of climate and adaptation (MCA–PD) causes a mean of 13,547 premature deaths (mean estimate range across studies: 1,320 to 26,481) based on the average of all functions (roughly the sum of climate alone and adaptation alone), with mean costs of $126 billion (mean estimate range across studies: $12 billion to $246 billion). The average 95% CI across studies is 2,685 to 26,213 deaths.

Considering the main focus of these results, the health impact of projected mid-century building energy use on PM_2.5_ (MCA–MCCO), we find, as stated above, a range of mean estimates of 87 to 1,245 excess deaths annually ($1 billion to $12 billion in costs), with an average of 654 deaths ($6 billion). For comparison, application of the C-R function from the most representative epidemiological study, the American Cancer Society’s Cancer Prevention Study II [89], finds a mean estimate of 366 (95% CI: 249 to 486) deaths annually, slightly on the lower end of all study estimates.

For O_3_, the results are similar to the findings for PM_2.5_, but additional functions address mortality from specific causes. The health impacts of projected mid-century building energy use on O_3_ (MCA–MCCO) include an average of 315 deaths ($3 billion) based on 3 standard configuration studies with a range of 198 to 438; ($2 billion to $4 billion). The average 95% CI across studies is 184 to 447 deaths. Using maximum daily 1-hour O_3_ concentrations to assess this same scenario (MCA–MCCO), one study calculates mortality from all causes, finding 369 additional deaths. Analyzing the studies with common health endpoints, we find that using maximum daily 1-hour O_3_ rather than MDA8 O_3_ concentrations results in higher mortality from all causes (369 versus 315 deaths annually) and more non-accidental deaths (189 versus 162 deaths annually).

For comparison of these building-related impacts with the health impacts associated with climate change alone (MCCO–PD), we calculated premature mortality from all causes and MDA8 O_3_ exposure as 3,234 deaths (range of 2,001 to 4,527) based on three studies and non-accidental mortality from MDA8 O_3_ exposure as 1,692 deaths (range of 888 to 4,194) based on 5 studies. Using maximum daily 1-hour concentrations, we find 4,431 all-cause deaths (range of 2,994 to 5,895) and 2,241 non-accidental deaths (range of 1,671 to 3,111). For MCA-PD, we calculated 3,514 deaths on average (range of 2,175 to 4,920), with a cost of $32.5 billion. The average 95% CI across studies is 2,028 to 5,026 deaths. Using MDA8 O_3_ and considering premature mortality from all causes, we find that 8.0% of additional deaths in the MCA scenario are from adaptation and 92.0% are from climate alone, i.e., adaptation yields an 8.7% increase above climate change impacts alone.

Morbidity impacts are summarized in S2–S4 Tables. Health impacts are assessed for endpoints including hospital admissions, respiratory symptoms (including asthma), minor restricted activity days, work loss days, and school loss days. Mean estimates of the costs of morbidity impacts vary from $0 to $45 million annually for PM_2.5_, $0 to $39 million annually for MDA8 O_3_, and $6 million to $18 million for maximum daily 1-hour O_3_.

**Fig 7:**
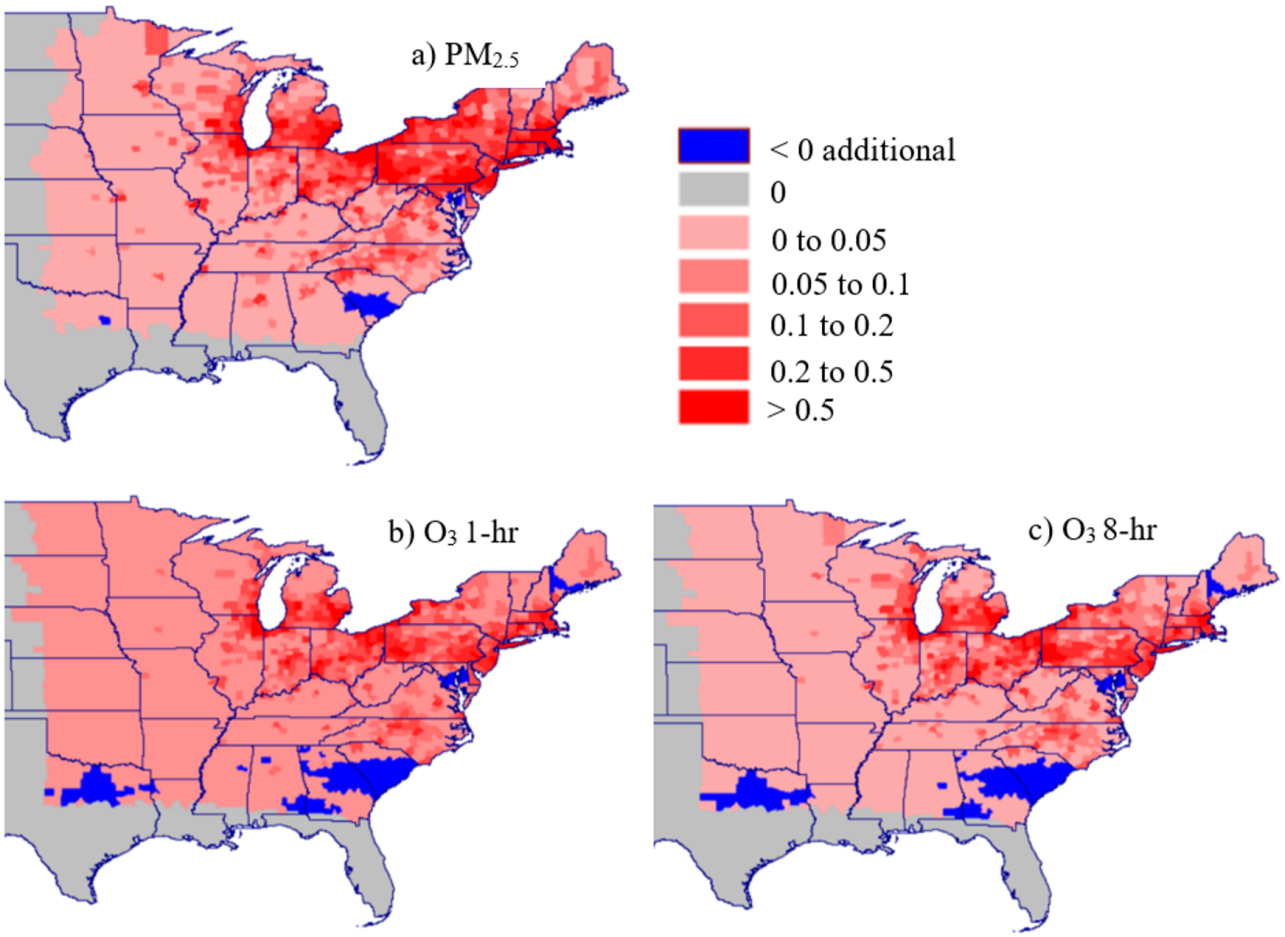
The mortality impacts of adaptation due to air pollution. Shown is the air-pollution-related mortality increase due to adaptation (the mid-century adaptation scenario minus the mid-century climate-only scenario) for (a) PM_2.5_ as taken from the Expert F concentration–response function (the median function), (b) O_3_ based on Levy et al. [97] using maximum daily 1-hour concentrations, and (c) O_3_ based on Levy et al. [97] using maximum daily 8-hour average concentrations.

The independent health impact estimates from exposure to PM_2.5_ and O_3_ cannot be directly summed because BenMAP does not account for interaction effects between the 2 pollutants, and exposures often occur in the same location at the same time. The spatial distributions of mortality are shown by county in Fig 7 for PM_2.5_ and O_3_ (maximum daily 1-hour and MDA8). The spatial distribution of impacts follows the patterns seen for air pollution in Fig 5. Regions near the Ohio River Valley and urban areas see the greatest mortality damages. In South Carolina, the Chesapeake Bay, and small portions of Maine and Texas, there is a slight decrease in mortality associated with a small, localized decrease in modeled emissions associated with modeled building energy demand and electricity dispatch.

## Discussion

Simulating adaptive behavior to a warmer mid-century climate shows that increased air conditioning use leads to higher emissions, degraded air quality, and adverse health outcomes. We find that the increase in air-pollution-related health outcomes attributable to climate change alone is 92%–95% of the overall health burden (depending on air pollutant), while changes in human behavior to adapt to climate change through increased air conditioning in buildings comprises 5%–8% of the health burden.

While our adaptation-related results are novel, our climate-only results are comparable to existing findings. Weaver et al. find that substantial regions of the US show increases in MDA8 O_3_ of 2–8 ppbv in a future climate [21], and Jacob and Winner find increases in O_3_ of 1–10 ppbv [20]. Fiore et al. find that previous studies show O_3_ increases of up to 9 ppbv [19]. For PM_2.5_, Jacob and Winner find an increase of 0.1 to 1 μg/m^3^ [20], and Fiore et al. find a greater variability of results across studies dependent upon meteorology, ranging from −2 to +3 μg/m^3^ [19]. Tai et al. find that PM_2.5_ likely will not increase by more than 0.5 μg/m^3^ [104]. Our findings fit within the high end of previous estimates, and this is expected as we consider a particularly warm July, when large increases in PM_2.5_ would be expected.

Quantifying the role of air conditioning adaptation in future air quality bears relevance to decision-making, as power sector emissions are controllable by technology and policy in a way that other climate-driven air quality mechanisms are not (i.e., chemical reaction rates, biogenic emissions, NO_X_ from lightning, and wildfire emissions). The scenario chosen here highlights the role of interactive effects amongst climate, energy production, and air quality. Interventions would, and likely will, reduce the damages calculated here. Control options include stack-level technological controls, such as SO_2_ scrubbers and NO_X_ selective catalytic reduction, which have been the traditional approach employed by US air quality management agencies and power sector utilities to meet health-based standards. Although this technological approach would serve to reduce pollution exposure, such strategies do not modulate cost, energy use, or carbon emissions. In fact, end-of-pipe controls increase energy requirements to balance the decrease in plant efficiency associated with effluent treatment methods; this is often called the capacity or heat rate penalty.

An alternative to end-of-pipe controls is the use of building energy efficiency measures (e.g., increasing insulation or installing more efficient cooling equipment [105,106]) that reduce building energy demand in a manner that directly responds to the increased utilization of air conditioning. Efficiency measures would reduce demand on the electricity system, as well as associated carbon emissions, air quality impacts, and adverse health outcomes. Another option to reduce both carbon emissions and air-pollution-related health impacts would be to increase the portion of electricity generated by renewable sources like solar and wind. Studies show that the use of solar energy would reduce and has reduced fine particulates in the eastern US, especially on the highest concentration days [39,107]. Other options include demand response programs, building codes and standards, and conservation education. All of these alternatives would mitigate climate change and reduce the air-pollution-related health burden from adaptation measures.

This study explores power plants and heat-driven electricity demand in buildings as an insufficiently understood mechanism of future air-quality-related health damages in a warmer climate. Here we parse the contribution of this adaptation, but the study limitations include modeling only a single representative month from 1 year in future climate projections. Typically, studies of climate would be based on a 30-year average of results, which is not computationally feasible for this type of study. Additionally, our results do not project future changes to population, air pollution exposure patterns in humans, building stock, and the electric power sector, but rather highlight the interactions amongst climate, electricity production, air quality, and health. With less computationally demanding methods, more simulations could be run over longer timeframes to test the sensitivity of results to potential changes. Future directions could also include assessing the impact of interventions for climate change mitigation and air pollution control. Lastly, health impacts assessment relies on C-R functions for O_3_ and PM_2.5_, and these relationships continue to be improved through epidemiological and toxicological research.

## Supporting information

S1 Fig The average summer temperatures of NARCCAP models and the present-day. (TIF)

S2 Fig Comparison of MyPower and NEI CMAQ results. (TIF)

S3 Fig Evaluation of the present-day simulations’ NO_2_ column amounts with satellite Ozone Monitoring Instrument NO_2_. (TIF)

S1 Table List of chemical species included in NEI emissions estimates from electricity generating units (EGUs). (DOCX)

S2 Table PM_2.5_-related morbidity results for standard configuration functions. (DOCX)

S3 Table MDA8 O_3_-related morbidity results for standard configuration functions. (DOCX)

S4 Table Maximum daily 1-hour O_3_-related morbidity results for included BenMAP functions. (DOCX)

S5 Table Validation of MyPower and CMAQ results. (DOCX)

S6 Table Measurement, model, and satellite correlations. (DOCX)

S7 Table Comparison of CMAQ NO_2_ results with DOMINO satellite NO_2_ estimates. (DOCX)

S1 Text A graphical depiction of temperatures from NARCCAP models shown in S1 Fig and referenced in the main text. (DOCX)

## Abbreviations

BenMAP: Environmental Benefits Mapping and Analysis Program
CB05: Carbon Bond 5
CCSM: Community Climate System Model
CMAQ: Community Multiscale Air Quality
C-R: concentration–response
EGU: electricity generating unit
EIA: Energy Information Administration
EPA: Environmental Protection Agency
MDA8: maximum daily 8-hour average
MEGAN: Model of Emissions of Gases and Aerosols from Nature
NAAQS: National Ambient Air Quality Standards
NARCCAP: North American Regional Climate Change Assessment Program
NARR: North American Regional Reanalysis
NEEDS: National Electric Energy Data System
NEI: National Emissions Inventory
NO_X_: nitrogen oxide(s)
ppbv: parts per billion by volume
RBESS: Regional Building Energy Simulation System
WRF: Weather Research and Forecasting
