# Supplementary figures and images for: Air-quality-related health impacts from climate change and from adaptation of cooling demand for buildings in the eastern United States: An interdisciplinary modeling study
Source: PLoS Med. 2018 Jul 3;15(7):e1002599. doi: 10.1371/journal.pmed.1002599 (PMC6029751; doi:10.1371/journal.pmed.1002599)

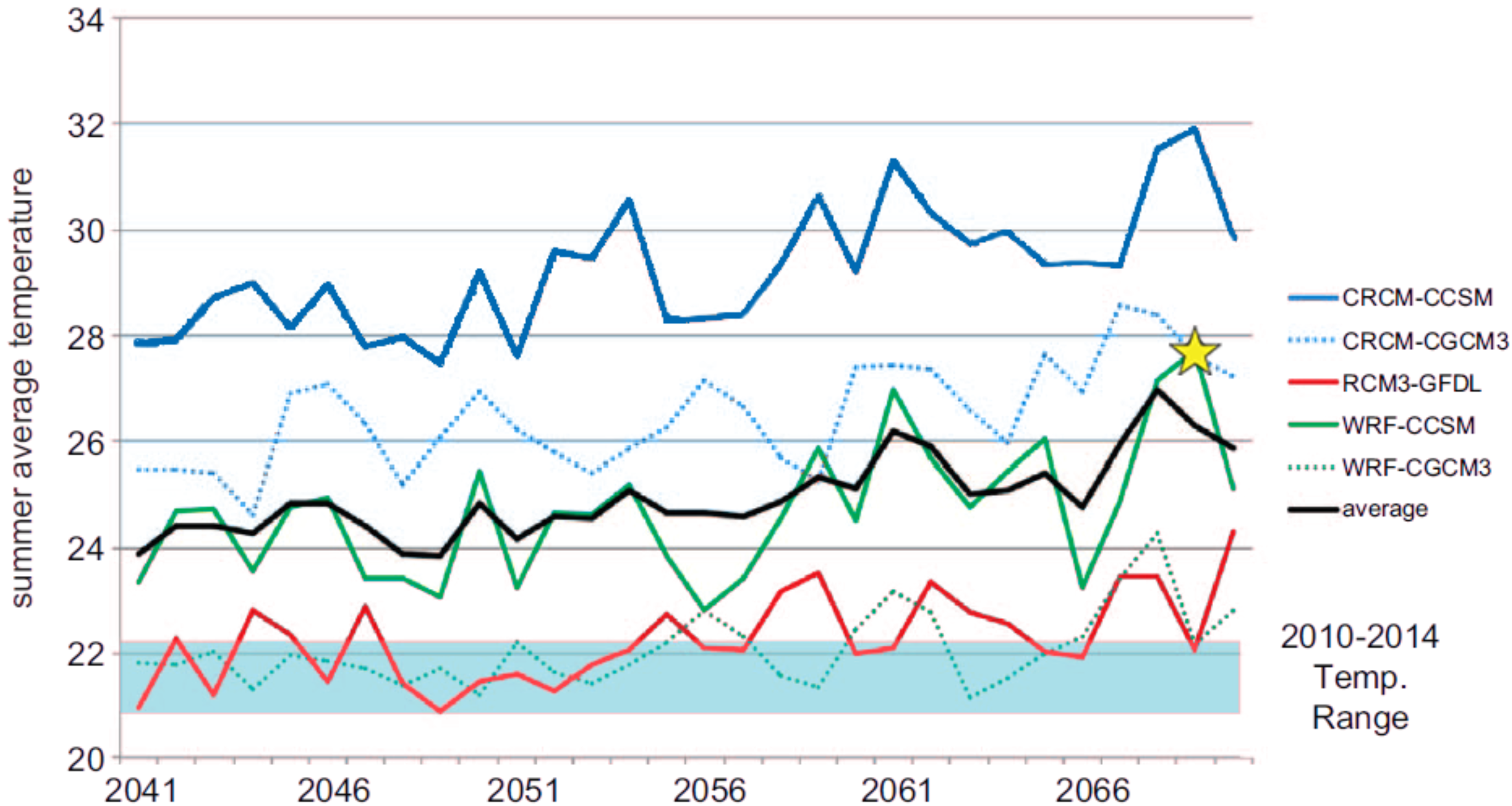

Supplement: S1 Fig — (TIF) [file pmed.1002599.s001.tif]

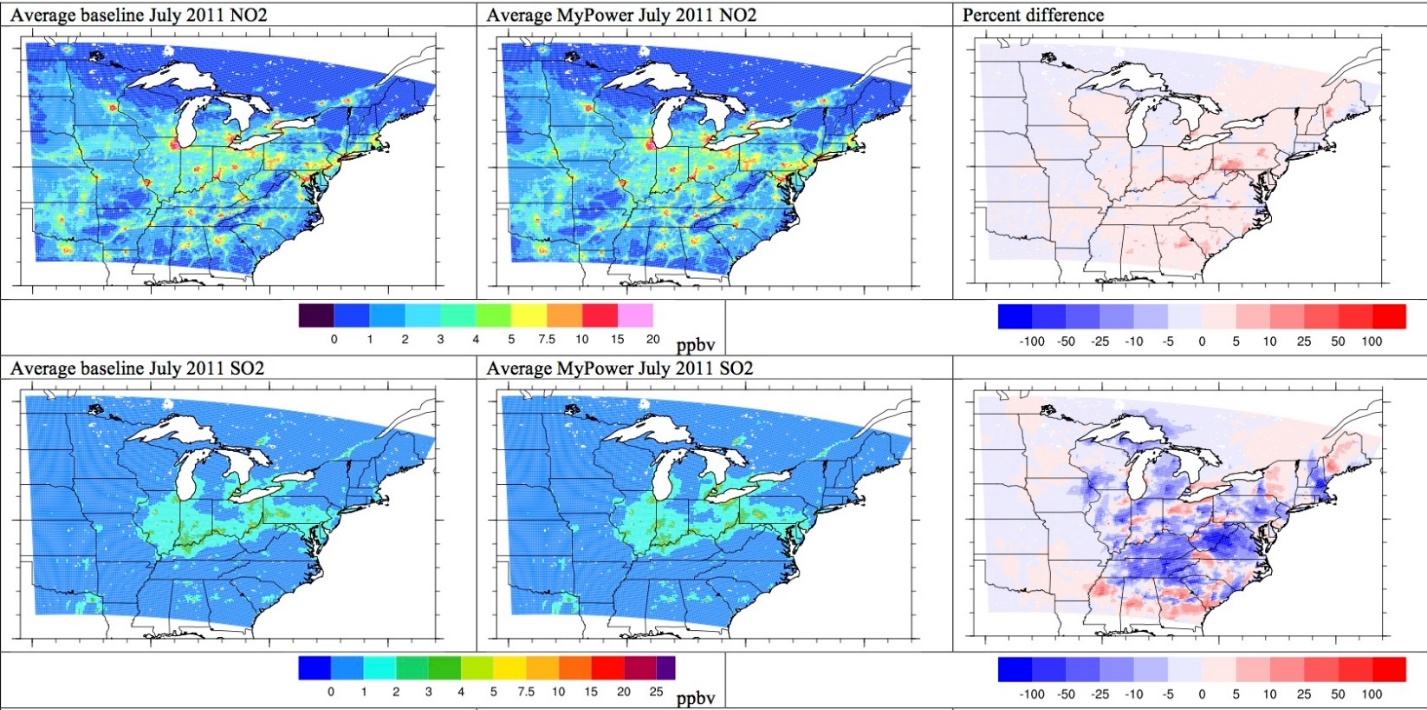

Supplement: S2 Fig — (TIF) [file pmed.1002599.s002.tif]

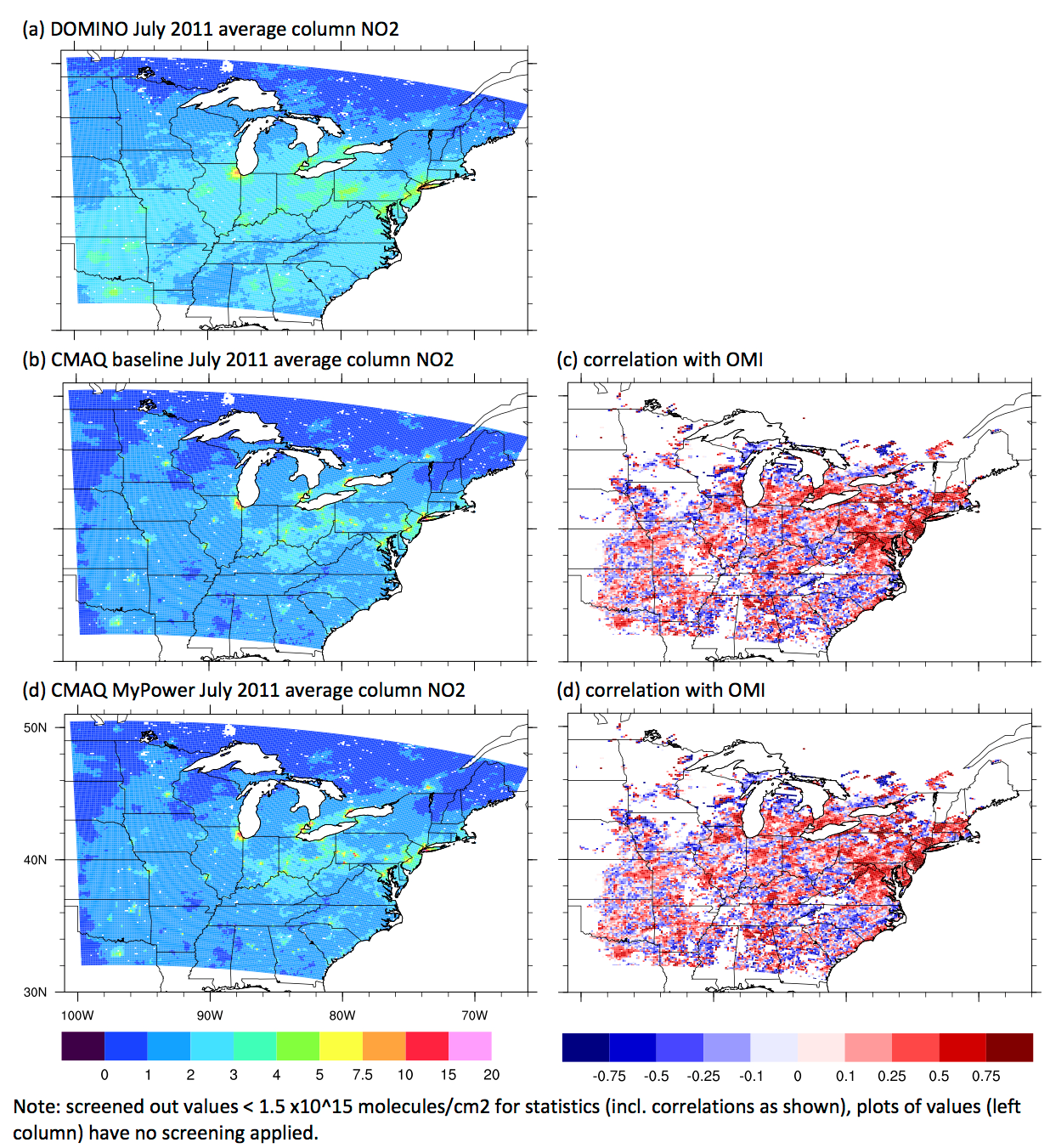

Supplement: S3 Fig — (TIF) [file pmed.1002599.s003.tif]
