# Supplementary material for: Air-quality-related health impacts from climate change and from adaptation of cooling demand for buildings in the eastern United States: An interdisciplinary modeling study
Source: PLoS Med. 2018 Jul 3;15(7):e1002599. doi: 10.1371/journal.pmed.1002599 (PMC6029751; doi:10.1371/journal.pmed.1002599)
Supplement: S1 Table — (DOCX) [file pmed.1002599.s004.docx]

| ALD2 | FORM_PRIMARY | OLE | PMOTHR | SULF |
| --- | --- | --- | --- | --- |
| ALD2_PRIMARY | HCL | PAL | PNA | TERP |
| ALDX | HONO | PAR | PNCOM | TOL |
| BENZENE | IOLE | PCA | PNH4 | UNK |
| CH4 | ISOP | PCL | PNO3 | UNR |
| CL2 | MEOH | PEC | POC | VOC_INV |
| CO | NH3 | PFE | PSI | XYL |
| ETH | NH3_FERT | PH2O | PSO4 |  |
| ETHA | NO | PK | PTI |  |
| ETOH | NO2 | PMG | PMC |  |
| FORM | NVOL | PMN | SO2 |  |

S1 Table. List of chemical species included in NEI emissions estimates from electricity generating units (EGUs).
