# Supplementary material for: Air-quality-related health impacts from climate change and from adaptation of cooling demand for buildings in the eastern United States: An interdisciplinary modeling study
Source: PLoS Med. 2018 Jul 3;15(7):e1002599. doi: 10.1371/journal.pmed.1002599 (PMC6029751; doi:10.1371/journal.pmed.1002599)
Supplement: S2 Table — (DOCX) [file pmed.1002599.s005.docx]

| PM_2.5_ 24-hr Mean | | | | MCA-MCCO | | MCCO-PD | | MCA-PD | |
| --- | --- | --- | --- | --- | --- | --- | --- | --- | --- |
| Health Outcome | C-R Function | Location | Age | Incidence (95% CI) | Valuation (million $) | Incidence (95% CI) | Valuation (million $) | Incidence (95% CI) | Valuation (million $) |
| Hospital Admissions, Asthma | Babin et al. Sheppard | N/A | 0-64 | -3  (-5, -1) | 0  (0, 0) | -64  (-105, -22) | -1  (-2, 0) | -67  (-109, -23) | -1  (-2, 0) |
| Hospital Admissions, All Respiratory | Kloog et al. Zanobetti et al | N/A | 65-99 | -17  (-34, 11) | -1  (-1, 0) | -353  (-724, 235) | -11  (-23, 7) | -370  (-759, 246) | -12  (-24, 8) |
| Hospital Admissions, Chronic Lung Disease | Moolgavkar | Los Angeles, CA | 18-64 | -8  (-3, -13) | 0  (0, 0) | -162  (-55, -270) | -3  (-5, -1) | -170  (-58, -283) | -3  (-6, -1) |
| Hospital Admissions, All Cardiovascular | Bell et al. Zanobetti et al Peng et al. | N/A | 65-99 | -17  (-34, -6) | -1  (-1, 0) | -342  (-699, -133) | -13  (-26, -5) | -359  (-733, -140) | -14  (-28, -5) |
| Hospital Admissions, All Cardiovascular | Moolgavkar | Los Angeles, CA | 18-64 | -12  (-6, -18) | 0  (-1, 0) | -263  (-137, -390) | -11  (-16, -6) | -275  (-143, -408) | -11  (-16, -6) |
| Acute Bronchitis | Dockery et al. | 24 communities | 8-12 | -163  (38, -364) | 0  (0, 0) | -3508  (816, -7869) | -2  (-5, 0) | -3673  (854, -8238) | -2  (-5, 0) |
| Emergency Room Visits, Asthma | Glad et al. Mar et al. Slaughter et al. | N/A | 0-99 | -64  (-139, 24) | 0  (0, 0) | -1452  (-3217, 523) | -1  (-1, 0) | -1518  (-3365, 547) | -1  (-2, 0) |
| Asthma Exacerbation, Wheeze | Ostro et al. Mar et al. | N/A | 6-18 | -3432  (-7908, 207) | 0  (-1, 0) | -73713  (-170330, 4403) | -4  (-12, 0) | -77177  (-178336, 4610) | -4  (-12, 0) |
| Work Loss Days | Ostro | Nationwide | 18-64 | -14918  (-12623, -17210) | -2  (-3, -2) | -323416  (-272456, -374642) | -50  (-58, -43) | -338761  (-285383, -392422) | -53  (-61, -45) |
| Minor Restricted Activity Days | Ostro and Rothschild | Nationwide | 18-64 | -89437  (-72816, -106048) | -6  (-9, -3) | -1967660  (-1588449, -2351078) | -133  (-203, -70) | -2061334  (-1664022, -2463088) | -139  (-212, -73) |
| Upper Respiratory Symptoms | Pope et al. | Utah Valley | 9-11 | -2997  (-544, -5448) | 0  (0, 0) | -64449  (-11630, -117631) | -2  (-5, 0) | -67470  (-12175, -123143) | -2  (-5, 0) |
| Lower Respiratory Symptoms | Schwartz and Neas | 6 U.S. cities | 7-14 | -2114 (-799, -3436) | 0 (0, 0) | -50519 (-17755, -86471) | -1 (-2, 0) | -52941 (-18591, -90688) | -1 (-2, 0) |
| Acute Myocardial Infarction Nonfatal | Peters et al. | N/A | 18-99 | -118  (-28, -208) | -15  (-37, -2) | -2838  (-613, -5398) | -361  (-965, -55) | -2980  (-643, -5676) | -379  (-1014, -57) |

S2 Table. PM_2.5_ morbidity results for standard configuration functions.

Values are annual impacts based on July exposure to exacerbated pollution.
