# Supplementary material for: Air-quality-related health impacts from climate change and from adaptation of cooling demand for buildings in the eastern United States: An interdisciplinary modeling study
Source: PLoS Med. 2018 Jul 3;15(7):e1002599. doi: 10.1371/journal.pmed.1002599 (PMC6029751; doi:10.1371/journal.pmed.1002599)
Supplement: S3 Table — (DOCX) [file pmed.1002599.s006.docx]

| MDA8 O_3_ | | | | MCA-MCCO | | MCCO-PD | | MCA-PD | |
| --- | --- | --- | --- | --- | --- | --- | --- | --- | --- |
| Health Outcome | Health Impact Function | Location | Age | Incidence (95% CI) | Valuation (mil. $) | Incidence (95% CI) | Valuation (mil. $) | Incidence (95% CI) | Valuation (mil. $) |
| Minor Restricted Activity Days | Ostro and Rothschild | Nationwide | 18-64 | -184548  (-75865,  -293657) | -13  (-23, -5) | -2277741  (-871897, -3819183) | -154  (-295,  -54) | -2462767  (-944720, -4123735) | -166  (-318,  -58) |
| Asthma Exacerbation: One or More Symptoms | Schildcrout et al. Mortimer et al. |  | 6-18 | -80025  (-195233, 68001) | -5  (-14, 4) | -1034688  (-2819467, 724076) | -60  (-191, 42) | -1132954  (-3070627, 797659) | -66  (-209, 46) |
| Emergency Room Visits: Asthma | Glad et al. Peel et al. Sarnat et al. Wilson et al. Ito et al. Mar and Koenig |  | 0-99 | -235  (-698, -22) | 0  (0, 0) | -2948  (-8689, -275) | -1  (-4, 0) | -3167  (-9314, -297) | -2  (-5, 0) |
| School Loss Days: All Cause | Chen et al. Gilliland et al. |  | 5-17 | -57995  (-132544,  -20664) | -6  (-13,  -2) | -580201  (-939950, -219929) | -56  (-90, -21) | -639569  (-1036129, -242433) | -61  (-99, -23) |
| Hospital Admissions: All Respiratory | Katsouyanni et al. | 14 U.S. cities | 65-99 | -45  (11, -101) | -1  (-3, 0) | -466  (105, -1066) | -15  (-34, 3) | -506  (114, -1157) | -16  (-37, 4) |

S3 Table: MDA8 O_3_ morbidity results for standard configuration functions.

Values are annual impacts based on July exposure to exacerbated pollution.
