# Supplementary material for: Air-quality-related health impacts from climate change and from adaptation of cooling demand for buildings in the eastern United States: An interdisciplinary modeling study
Source: PLoS Med. 2018 Jul 3;15(7):e1002599. doi: 10.1371/journal.pmed.1002599 (PMC6029751; doi:10.1371/journal.pmed.1002599)
Supplement: S4 Table — (DOCX) [file pmed.1002599.s007.docx]

| Daily 1-hr Max O_3_ | | | | MCA-MCCO | | MCCO-PD | | MCA-PD | |
| --- | --- | --- | --- | --- | --- | --- | --- | --- | --- |
| Health Outcome | Health Impact Function | Location | Age | Incidence (95% CI) | Valuation (mil. $) | Incidence (95% CI) | Valuation (mil. $) | Incidence (95% CI) | Valuation (mil. $) |
| Hospital Admissions: All Respiratory | ^a^Burnett et al. | Toronto, CAN | 0-1 | -71  (-30, -112) | -2  (-3, -1) | -1290  (-423, -2489) | -39  (-75, -13) | -1381  (-455, -2657) | -41  (-80, -14) |
| School Loss Days: All Cause | ^a^Chen et al. | Washoe Co, NV | 5-17 | -52432  (-13683,  -91124) | -5  (-9, -1) | -622737  (-162510, -1082300) | -61  (-106, -16) | -675168  (-176192, -1173417) | -66  (-115, -17) |
| Minor Restricted Activity Days | ^a^Ostro and Rothschild | Nationwide | 18-64 | -179086  (-73566,  -285123) | -6  (-14, 0) | -2470003  (-943868, -4147846) | -78  (-197, -3) | -2661779  (-1019303, -4463587) | -84  (-213, -4) |

S4 Table. Daily Maximum 1-hr O_3_ morbidity results for included BenMAP functions.

Values are annual impacts based on July exposure to exacerbated pollution.
