# Supplementary material for: Air-quality-related health impacts from climate change and from adaptation of cooling demand for buildings in the eastern United States: An interdisciplinary modeling study
Source: PLoS Med. 2018 Jul 3;15(7):e1002599. doi: 10.1371/journal.pmed.1002599 (PMC6029751; doi:10.1371/journal.pmed.1002599)
Supplement: S5 Table — (DOCX) [file pmed.1002599.s008.docx]

|  | Baseline 2011 average value | MyPower 2011 average value | Average percent diff. | RMSE | Mean Error | Mean Fractional Error | Mean Bias | Mean Fractional Bias | Correlation (r^2^) |
| --- | --- | --- | --- | --- | --- | --- | --- | --- | --- |
| NO_2_ | 1.916 | 1.930 | 0.728 | 0.052 | 0.032 | 1.478 | 0.014 | 0.600 | 0.993 |
| SO_2_ | 0.572 | 0.569 | -0.537 | 0.164 | 0.103 | 12.172 | -0.003 | -1.611 | 0.955 |

S5 Table. Validation of MyPower and CMAQ results.

Calculated using baseline 2011 as “truth”, all stats are for gridpoints on land only (shown above). Average % difference calculated using averages given in this table rather than average of % difference for each gridpoint.
