# Supplementary material for: Air-quality-related health impacts from climate change and from adaptation of cooling demand for buildings in the eastern United States: An interdisciplinary modeling study
Source: PLoS Med. 2018 Jul 3;15(7):e1002599. doi: 10.1371/journal.pmed.1002599 (PMC6029751; doi:10.1371/journal.pmed.1002599)
Supplement: S6 Table — (DOCX) [file pmed.1002599.s009.docx]

|  |  | **Baseline 2011** | **MyPower 2011** |  | **Baseline  2011** | **MyPower 2011** |
| --- | --- | --- | --- | --- | --- | --- |
|  | **AQS** | **CMAQ-SFC** | **CMAQ-SFC** | **DOMINO** | **CMAQ-COL** | **CMAQ-COL** |
| **PBLH** | -0.144 | -0.221 | -0.231 | -0.026 | -0.100 | -0.099 |
| **WINDSP** | -0.028 | -0.049 | -0.068 | -0.005 | -0.020 | -0.022 |
| **INSOL** | -0.125 | -0.179 | -0.144 | -0.070 | -0.125 | -0.123 |
| **RH** | 0.153 | 0.126 | 0.128 | 0.074 | 0.236 | 0.234 |
| **PRECIP** | 0.107 | 0.078 | 0.071 | 0.030 | 0.267 | 0.265 |
| **T** | 0.397 | 0.203 | 0.129 | 0.179 | 0.223 | 0.218 |
| **SLP** | -0.183 | -0.229 | -0.176 | -0.132 | -0.136 | -0.134 |

S6 Table. Measurement, Model, and Satellite Correlations.

July 2011 averaged correlations between Air Quality System (AQS) measurements, CMAQ runs, and Dutch Ozone Monitoring Instrument NO_2_ (DOMINO) for meteorological variables over the Eastern U.S. corresponding to satellite overpass time (1 pm LST). Correlation values using surface-layer CMAQ data are constrained to grid cells co-located with AQS data.
