# Supplementary material for: Air-quality-related health impacts from climate change and from adaptation of cooling demand for buildings in the eastern United States: An interdisciplinary modeling study
Source: PLoS Med. 2018 Jul 3;15(7):e1002599. doi: 10.1371/journal.pmed.1002599 (PMC6029751; doi:10.1371/journal.pmed.1002599)
Supplement: S7 Table — (DOCX) [file pmed.1002599.s010.docx]

|  | July 2011 average | RMSE | Mean error | Mean bias | Mean fractional error | Mean fractional bias | r2 |
| --- | --- | --- | --- | --- | --- | --- | --- |
| DOMINO | 2.627 |  |  |  |  |  |  |
| CMAQ baseline | 2.565 | 1.280 | 0.505 | -0.150 | 17.007 | -6.871 | 0.076 |
| CMAQ MyPower  (PD scenario) | 2.601 | 1.287 | 0.515 | -0.136 | 17.188 | -6.563 | 0.075 |

S7 Table. Comparison of CMAQ NO_2_ results with DOMINO (satellite NO_2_).
