# Supplementary material for: Air-quality-related health impacts from climate change and from adaptation of cooling demand for buildings in the eastern United States: An interdisciplinary modeling study
Source: PLoS Med. 2018 Jul 3;15(7):e1002599. doi: 10.1371/journal.pmed.1002599 (PMC6029751; doi:10.1371/journal.pmed.1002599)
Supplement: S1 Text — (DOCX) [file pmed.1002599.s011.docx]

**S1 Text**

A graphical depiction of temperatures from NARCCAP models are shown in S1 Fig and referenced in the main text.

S1 Fig. The average summer temperatures of NARCCAP models and the present day.

Adapted from a figure in Meier et al., (2017) [1]. The year chosen for analysis is represented by the yellow star. It is the hottest year from the best performing model. A range of summer average temperatures from 2010–2014 is shown by the blue band.

*Evaluation of simulated emissions and air quality*

We evaluate MyPower emissions using present-day (2011) simulations of NO_2_ and SO_2_, as they are directly emitted by EGUs and will highlight differences in emissions inputs. For evaluation, one present-day simulation employs all anthropogenic emissions from the 2011 NEI, while the second (PD scenario) substitutes EGU emissions of NO_X_ and SO_2_ from the MyPower system of models for NEI EGU emissions, as described in the main text. Emissions of species shown in S1 Table are assigned NEI regional averages on an hourly basis. Results show small differences in NO_2_ and SO_2_ concentrations when EGU emissions from the NEI and MyPower system of models are used. These differences are shown in S2 Fig and summarized in S5 Table. As shown in S2a Fig and S2b Fig, near-surface NO_2_ amounts are higher in densely populated areas and in transportation corridors, reflecting sources from vehicular combustion and power generation. Modeling with MyPower EGU emissions results in NO_2_ with a mean bias of 0.014 ppbv (~0.7%) compared to the present-day simulation using NEI EGU emissions (S5 Table). As seen in S2c Fig, concentrations of NO_2_ are higher in the MyPower simulation by 5-10% in some areas, including the Ohio River Valley and south-central Pennsylvania.

S2 Fig. Comparison of MyPower and NEI CMAQ Results.

Comparison of surface NO_2_ and SO_2_ concentrations with emissions simulated using MyPower as compared to the U.S. EPA’s National Emissions Inventory.

With a high concentration of fossil-fuel fired power plants in the Ohio River Valley, NO_X_ emissions from EGUs in Pennsylvania, Indiana, and Ohio are greater than EGU NO_X_ emissions from any other state in the Eastern U.S., in both the NEI and MyPower EGU emissions datasets. We expect some spatial heterogeneity in emissions between these datasets, however, given potential differences in the energy demand as simulated by RBESS and differences in power plant characteristics Meier et al., (2017), as well as our partitioning of total NO_X_ output from the MyPower system to NO and NO_2_.

Given these small differences in near-surface amounts of NO_2_, which rely heavily on emissions, and that both simulations employ the same meteorological data, the relationships between NO_2_ estimates and meteorological variables in both present-day simulations are very similar, and follow NO_2_-climate patterns described (Harkey et al., 2015) (S6 Table); in line with observed relationships, model simulations show positive relationships with relative humidity, precipitation, and temperature, and negative relationships with horizontal wind, insolation, sea level pressure, and boundary layer height. Additionally, both present-day simulations' column NO_2_ amounts compare similarly to satellite-based observations of column NO_2_ (S3 Fig, S7 Table), with model simulations capturing the overall pattern of urban maxima, but overestimating column NO_2_ over urban areas and underestimating amounts over the rest of the Eastern U.S., consistent with past work (e.g. Harkey et al., 2015 [2]).

S3 Fig. Evaluation of present-day simulations’ NO_2_ column amounts with satellite, OMI NO_2_.

Sulfur dioxide amounts are greatest in the Ohio River valley, the location of many coal-fired power plants in both present-day simulations--up to 10 ppbv (S2dFig, S2e Fig). Differences in SO_2_ amounts between present-day simulations are less than those for NO_2_ on average: the mean bias of the simulation using MyPower emissions for SO_2_ is -0.003 ppbv (~ -0.5%, S6 Table). However, the variance is greater than seen for NO_2_, with percent differences in SO_2_ ranging from -25% to 25% (S2f Fig). These differences in SO_2_, as with the differences in NO_2_ amounts simulated by both present-day scenarios, are caused by differences in EGU emissions modeling. Overall, MyPower emissions of SO_2_ in the region are 2.8% lower than those from the NEI, and in states with the largest SO_2_ emissions—Ohio, Indiana, and Pennsylvania—emissions are 25.5% greater, 6.3% lower, and 20.6% greater in the MyPower emissions dataset respectively. Proportionally large decreases in SO_2_ emissions relative to the NEI dataset also exist in Kentucky (-12.4%) and Tennessee (-39.1%), and the locations of these lower emissions correspond to locations with lower SO_2_ concentrations shown in S2f Fig.

*How to Access Software and Procedures*

*Weather Research and Forecasting Model (WRF)*: WRF is a publicly available model developed in part and managed by the National Center for Atmospheric Research and University Corporation for Atmospheric Research. It is available online through their sites, <https://www.mmm.ucar.edu/weather-research-and-forecasting-model>.

*North American Regional Climate Change Assessment Project (NARCCAP) – Community Climate System Model (CCSM):* Data from the North American Regional Climate Change Assessment Project is available through the University Corporation for Atmospheric Research at the site: <http://www.narccap.ucar.edu/>.

*Regional Building Energy Simulation System (RBESS)*: RBESS was developed for the purposes of this study by Seventhwave, led by author Doug Ahl. The system is a batch modeling procedure based on a publicly available modeling platform, DOE-2, developed by developed by [James J. Hirsch & Associates (JJH)](mailto:Jeff.Hirsch@DOE2.com) in collaboration with [Lawrence Berkeley National Laboratory (LBNL)](http://www.lbl.gov/) primarily funded by the U.S. Department of Energy and currently managed by JJH at the site <http://doe2.com/>. For requests regarding RBESS contact author Doug Ahl at Seventhwave directly.

*MyPower electricity dispatch and emissions model*: The MyPower model is a proprietary model developed by study author Paul Meier and owned by Meier Engineering Research, LLC. MyPower is made accessible at: <https://juicebox.org/>. However the data used in this study is publicly available.

*Community Multiscale Air Quality (CMAQ) model*: CMAQ is a publicly available model, developed and managed by the U.S. Environmental Protection Agency. For information regarding CMAQ visit: <https://www.epa.gov/cmaq>.

*Environmental Benefits Mapping and Analysis Program (BenMAP):* BenMAP is a publicly available model, developed and managed by the U.S. Environmental Protection Agency. For information regarding BenMAP visit: <https://www.epa.gov/benmap>.

S1 Text References:

1. Meier P, Holloway T, Patz J, Harkey M, Ahl D, Abel D, et al. Impact of warmer weather on electricity sector emissions due to building energy use. Environ Res Lett. 2017;12: 064014. doi:[10.1088/1748-9326/aa6f64](https://doi.org/10.1088/1748-9326/aa6f64)

2. Harkey M, Holloway T, Oberman J, Scotty E. An evaluation of CMAQ NO2 using observed chemistry-meteorology correlations. Journal of Geophysical Research: Atmospheres. 2015; 1–19. doi:[10.1002/2014JD022994.Received](https://doi.org/10.1002/2014JD022994.Received)
